# The origin of the solvent dependence of fluorescence quantum yields in dipolar merocyanine dyes†
†Dedicated to Prof. Michael R. Wasielewski on the occasion of his 70th birthday.
‡
‡Electronic supplementary information (ESI) available. CCDC 1957268. For ESI and crystallographic data in CIF or other electronic format see DOI: 10.1039/c9sc05012d


**DOI:** 10.1039/c9sc05012d

**Published:** 2019-12-04

**Authors:** Joscha Hoche, Alexander Schulz, Lysanne Monika Dietrich, Alexander Humeniuk, Matthias Stolte, David Schmidt, Tobias Brixner, Frank Würthner, Roland Mitric

**Affiliations:** a Institut für Physikalische und Theoretische Chemie , Universität Würzburg , Am Hubland , 97074 Würzburg , Germany . Email: brixner@phys-chemie.uni-wuerzburg.de ; Email: roland.mitric@uni-wuerzburg.de; b Institut für Organische Chemie , Universität Würzburg , Am Hubland , 97074 Würzburg , Germany . Email: wuerthner@uni-wuerzburg.de; c Center for Nanosystems Chemistry & Bavarian Polymer Institute (BPI) , Universität Würzburg , Theodor-Boveri-Weg , 97074 Würzburg , Germany

## Abstract

Fluorophores with high quantum yields are desired for a variety of applications. Optimization of promising chromophores requires an understanding of the non-radiative decay channels that compete with the emission of photons. We synthesized a new derivative of the famous laser dye 4-dicyanomethylen-2-methyl-6-*p*-dimethylaminostyryl-4*H*-pyran (**DCM**), *i.e.*, merocyanine 4-(dicyanomethylene)-2-*tert*-butyl-6-[3-(3-butyl-benzothiazol-2-ylidene)1-propenyl]-4*H*-pyran (**DCBT**). We measured fluorescence lifetimes and quantum yields in a variety of solvents and found a trend opposite to the energy gap law. This motivated a theoretical investigation into the possible non-radiative decay channels. We propose that a barrier to a conical intersection exists that is very sensitive to the solvent polarity. The conical intersection is characterized by a twisted geometry which allows a subsequent photoisomerization. Transient absorption measurements confirmed the formation of a photoisomer in unpolar solvents, while the measurements of fluorescence quantum yields at low temperature demonstrated the existence of an activation energy barrier.

## Abstract 2

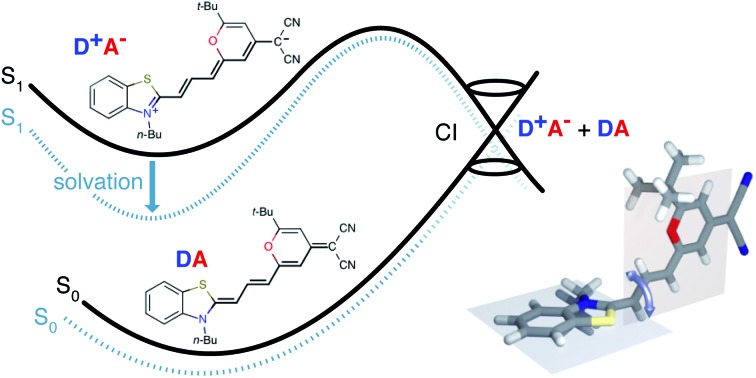
An increasing activation energy barrier to a conical intersection was identified as the reason for higher fluorescence lifetimes and quantum yields for merocyanines in polar solvents.

## 1 Introduction

Understanding and predicting photophysical and electronic properties of fluorophores are a prerequisite for many applications including biomolecular imaging,^1^ organic lasers,^2^ and light-emitting molecular devices.^3^,^4^ The usefulness of many organic fluorophores depends directly on the fluorescence quantum yield, which is defined by the competition of fluorescence to all other non-radiative relaxation channels. The ability to fluoresce is usually linked to a rigid, conjugated molecular structure which ensures that the excited state has a stable minimum.^5^–^7^ If the emissive geometry differs only little from the ground-state geometry, sharp and structured absorption and emission spectra are observed, which are approximately mirror images of each other.^5^ In contrast to that, non-fluorescent chromophores have reactive excited states and decompose or decay to the ground state non-radiatively before a photon can be emitted.^8^ The non-radiative decay occurs *via* crossing potential energy surfaces (PES) and drastic deformations of the geometry.^9^–^14^ Due to the fast nature of the decay, spectra are usually broad and featureless. This dichotomy of stable, fluorescent and reactive, non-fluorescent chromophores is also reflected in the theoretical modeling of those compounds: when fluorescence is dominant, the harmonic approximation is invoked and transitions (both radiative and non-radiative) between the harmonic potential energy surfaces of the initial and final electronic states are accounted for by perturbation theory.^15^–^18^ On the other hand, photochemical reactions require different approaches such as non-adiabatic molecular dynamics simulations, where the nuclei are treated as classical particles rolling on and jumping between potential energy surfaces.^19^–^24^ Such simulations, however, are limited to short timescales, not more than a few hundred femtoseconds, while typical experimental fluorescence lifetimes are in the order of nanoseconds. In recent years it has been noted that the distinction between stable and reactive chromophores is not that clear. Conical intersection can also play a role in non-ultrafast settings.^25^–^27^ In a recent data survey on distyrylbenzene derivatives an inverted energy gap law for the non-radiative decay rate was attributed to access to a conical intersection.^28^ Conical intersections in the vicinity of the Franck–Condon point may impact the fluorescence rate by opening an additional temperature-dependent channel for relatively slow non-radiative decay^29^–^31^ and photoisomerization.^12^,^32^–^35^ If a barrier separates a stable minimum close to the Franck–Condon point from a conical intersection, it is legitimate to assume that the reactions in the excited state are also governed by the rules of thermodynamics, so that one can apply transition-state theory for estimating the temperature-dependent rates of non-radiative decay.^25^,^36^ The energetic position of the barrier and conical intersection can be tuned by substitutions as demonstrated for naphthalene derivatives.^27^ Experimentally this situation is evidenced by the temperature dependence of the non-radiative rate.

**Fig. 1:**
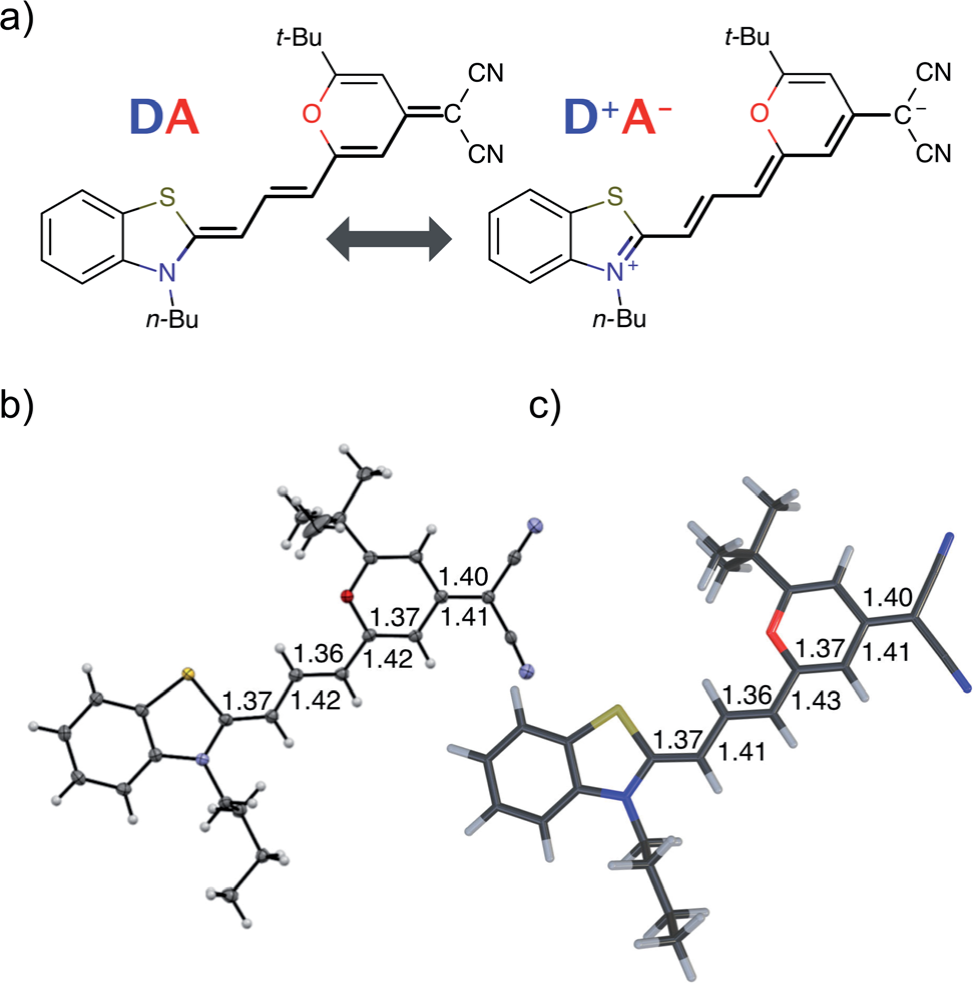
**DCBT** structure. (a) Neutral |**DA**〉 and zwitterionic | and zwitterionic |**D^+^A^–^**〉 resonance structures of merocyanine dye resonance structures of merocyanine dye **DCBT**. The polymethine chain connecting donor **D** and acceptor **A** is highlighted. (b) Molecular structure obtained from crystallographic analysis (thermal ellipsoids are set at 50% probability) (c) DFT calculations using IEFPCM solvation (in DMSO). The bond distances (in Å) along the polymethine chain are shown next to each bond.

In this contribution we study the photochemistry of a fluorescent merocyanine dye, 4-(dicyanomethylene)-2-*tert*-butyl-6-[3-(3-butyl-benzothiazol-2-ylidene)1-propenyl]-4*H*-pyran (**DCBT**, structure in Fig. 1), whose fluorescence quantum yield depends markedly on the polarity of the solvent and temperature.

Merocyanines^37^ are push–pull chromophores, which consist of a donor and an acceptor group that are covalently connected by a polymethine chain, which contains an odd number of –CH= groups.^38^ The optical properties of merocyanines can be tuned by the length of the polymethine chain and the strength of the donor and acceptor groups.^39^,^40^ Indeed, the choice of the donor/acceptor groups defines the electronic character that can span the range from neutral to zwitterionic.^40^–^48^ These remarkable characteristics are of large technological interest for the development of organic photovoltaics, nonlinear optics, and electronics.^49^–^52^

With regard to applications depending on fluorescence, however, merocyanines are difficult chromophores. Thus, despite of a polymethine chain that can be tuned to the cyanine limit by proper choice of donor and acceptor groups,^43^,^51^ the emissive properties of merocyanines are in general inferior to those of cyanine relatives. Thus, there are only a small number of merocyanine dyes such as the 4-dicyanomethylene-2-methyl-6-*p*-dimethylaminostyryl-4*H*-pyran laser dye **DCM**^53^ that show high fluorescence quantum yields in well-chosen solvents.^54^ In merocyanines as well as in cyanines solvent dependencies of the quantum yields were observed and possible relaxation channels were discussed.^12^,^31^,^54^–^56^ In this work we aim to elucidate the reason for this behavior of merocyanines. Our hypothesis is that the structural relaxation that follows the displacement of the electron cloud in these push–pull chromophores after photoexcitation might be hindered by a barrier to a conical intersection that is very sensitive to the polarity of the solvent. The presence of an accessible conical intersection can thus be probed by the variation of the fluorescence quantum yield with the dielectric constant of the solvent. Tuning the dielectric constant allows to gradually turn off the non-radiative decay channel through the conical intersection.

To evaluate this hypothesis we measured fluorescence quantum yields and built a theoretical model to understand the different relaxation channels of the merocyanine **DCBT** for the following series of solvents ordered by increasing dielectric constant (*ε*_r_): methylcyclohexane (MCH) < toluene (Tol) < chloroform (CHCl_3_) < dichloromethane (CH_2_Cl_2_) < acetonitrile (MeCN) < dimethylsulfoxide (DMSO).

## 2 Results

### Structural properties

Fig. 1 shows the molecular structure obtained from single-crystal X-ray analysis together with one of the optimized ground-state structures from DFT calculations of the merocyanine **DCBT** (for details on the chemical synthesis, ^1^H NMR, ^13^C and ROESY NMR as well as crystallographic data see ESI‡). It consists of a tertiary amine, which can donate a free electron pair, connected by a polymethine chain to a dicyanovinyl group, which can accept electrons. The acceptor half of the molecule is the same as in the DCM merocyanine laser dye while the donor group is part of a strongly electron-donating benzothiazolidene methylene base.

In the solid state **DCBT** shows an all-*trans* polymethine chain which is also the configuration observed in solution according to our ROESY-NMR studies (*cf.* Fig. S4 in the ESI‡). The C–C bond length alternation observed for the polymethine chain in the single crystal (Fig. 1b) indicates that the **DA** chromophore exhibits a more polyene-type structure with a dominating non-polar resonance structure (**DA** in Fig. 1a). This geometry also prevails in solution according to our electrooptical absorption measurements in methylcyclohexane (EOAM, see Fig. S6, ESI‡).

The merocyanine admits a neutral structure **DA** and a zwitterionic resonance structure **D^+^A^–^** (depicted in Fig. 1a), which are responsible for the solvatochromism. The resonance structures are constructed by constraining an electron to either the donor or the acceptor region. The ground and excited states are linear combinations of these two diabatic states,
1

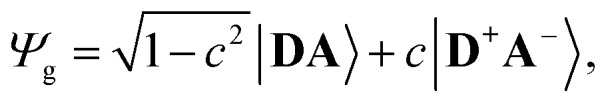



2

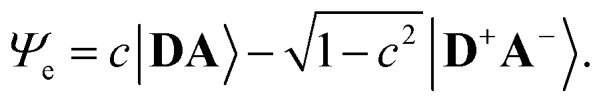




The resonance parameter *c*^2^ quantifies the character of the ground/excited-state wavefunction as neutral/zwitterionic (*c*^2^ = 0) or zwitterionic/neutral (*c*^2^ = 1).^43^,^49^ In between lies the cyanine limit *c*^2^ = 0.5 at which both diabatic states have equal weights. From electrooptical absorption measurements^57^,^58^ we find that the ground-state dipole moment is |*μ*(S_0_)| = 10.4 ± 0.6 D, suggesting that the neutral form dominates as expected in methylcyclohexane. In the first excited state the permanent dipole moment increases to |*μ*(S_1_)| = 19.9 ± 1.1 D, so that this state can be described by the zwitterionic resonance structure (**D^+^A^–^**). From the changes in the dipole moment it is possible to determine the weight of the zwitterionic configuration that is about *c*^2^ = 0.27 ± 0.02 for the ground state of **DCBT**.

### Optical properties

**Fig. 2:**
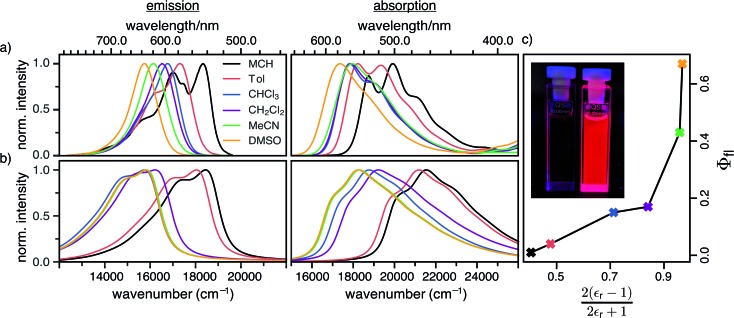
(a) Experimental absorption and emission spectra of **DCBT** in six different solvents. (b) Simulated vibrationally resolved absorption and emission spectra in the framework of TD-ωB97XD/def2-TZVP in combination with a polarizable continuum model using the integral equation formalism variant (IEFPCM). All simulated spectra were shifted by 0.3 eV to lower energies for better comparability to the experimental ones. (c) Dependence of the experimental fluorescence quantum yield on the dielectric screening factor of the solvent. The inset shows the optical photograph of samples (*c* = 10^–5^ M) in MCH (left) and DMSO (right) under black light illumination.

The steady-state absorption and fluorescence spectra of the **DCBT** merocyanine dye in the different solvents are depicted in Fig. 2a and the simulated spectra are shown in Fig. 2b. The spectral features of the dyes exhibit a large positive solvatochromism, both in absorption and fluorescence spectra. Thus, the absorption maxima shift from 534 nm in MCH to 576 nm in DMSO which is in accordance with the increase in dipole moment upon optical excitation, *i.e.*, stabilization of the more dipolar excited state by polar solvents. The bathochromic shift upon increasing solvent polarity is even larger in the fluorescence spectra, *i.e.*, from 546 nm in MCH to 637 nm in DMSO, correlating to an increase in the Stokes shift from 400 to 1700 cm^–1^. The most interesting result for this study is, however, the strong increase of fluorescence quantum yield upon increasing solvent polarity, *i.e.*, from ≈1% in MCH to 67% in DMSO (see Fig. 2c and Table 1).

**Table 1:** Theoretical (th.) and experimental (exp.) data for the characterization of the spectroscopic and electronic properties of the merocyanine **DCBT** in solvents of different polarity. See text for definitions of all quantities

Solvent	Δ*E*/eV	|*μ*_eg_|/D		*C*/eV	*E*_A_/eV	*γ* ^*a*^/cm^–1^	*ω*_*a*_/cm^–1^		*ω*_*b*_/cm^–1^		*k*_r_/10^8^ s^–1^		*k*_nr_/10^8^ s^–1^			*τ*_fl_/ns	Φ_fl_/%	
		Exp.	Th.				TS_1_	TS_2_	TS_1_	TS_2_	Exp.^*b*^	Th.	Exp.^*b*^	Theory		Exp.	Exp.	Th.
														*k* harm ic	*k* CI ic			
MCH	2.69	9.73	14.0	0.60	0.30	1047	117	210	344	401	>0.7^*c*^	10.5	>71^*c*^	0.0012	27 600	<0.2	1	0
Tol	2.65	9.04	14.2	0.66	0.32	845	117	228	359	395	1.8	11.4	44	0.0053	14 000	0.22	4	0.1
CHCl_3_	2.50	9.73	14.8	0.81	0.42	566	116	228	386	362	2.5	9.70	14	0.18	557	0.59	15	2
CH_2_Cl_2_	2.42	10.8	15.2	0.88	0.48	573	117	231	395	225	1.9	9.02	9.3	0.52	41.4	0.89	17	18
MeCN	2.34	9.78	15.5	0.98	0.58	886	116	230	363	181	2.4	7.56	3.2	5.38	0.80	1.78	43	55
DMSO	2.34	11.1	15.5	0.99	0.59	3641	116	230	333	175	2.9	9.18	1.4	6.05	0.22	2.32	67	60

^*a*^Details for the calculation of the friction coefficients are shown in ESI.

^*b*^Obtained from TCSPC and fluorescence quantum yield measurements.

^*c*^These radiative and non-radiative rate constants are only a lower limit, as the fluorescence lifetime is below the instrument response time of the TCSPC setup.

## 3 Discussion

The fluorescence quantum yield (Φ_fl_) is defined as the ratio between the radiative rate (*k*_r_) and all other rates including the non-radiative relaxation channels (*k*_nr_):
3

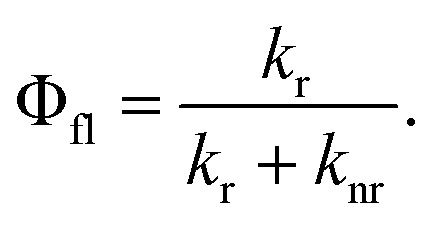




Radiative and non-radiative rates show opposite trends with excitation energy, when initial and final states are approximated by harmonic potential energy surfaces. The radiative rate increases as the third power of the emission energy, while the non-radiative rate decreases faster than exponentially as the excitation energy increases. Accordingly the fluorescence quantum yield should go up with the emission energy. However this is not consistent with the observations for **DCBT**, where the lowering of the emission energy due to the solvatochromic shift is accompanied by an increase in the fluorescence quantum yield.

The missing piece is an additional non-radiative deactivation channel that leads to a conical intersection (CI). We have to amend our model by a temperature-dependent rate for leaving the metastable minimum over a barrier. The non-radiative rate is thus split into two parts,

4 *k*_nr_ = *k*harmic + *k*CIic,

a temperature-independent rate that is calculated in the harmonic approximation with some additional simplifications (*k*harmic) and a temperature-dependent rate based on Kramers' theory (*k*CIic). The relaxation to triplet states is neglected in this work, as the experimental observation of fluorescence on the (sub)nanosecond timescale makes this relaxation pathway very unlikely. However, we cannot exclude that intersystem crossing contributes also to some degree to the relaxation channels.

### Radiative rate

The radiative rate increases as the second power of the refractive index *n* (see ref. 59 for a discussion of the refractive index correction), as the second power of the transition dipole moment *μ*_eg_, and as the third power of the emission energy, Δ*E* – *E*_**m**_:
5

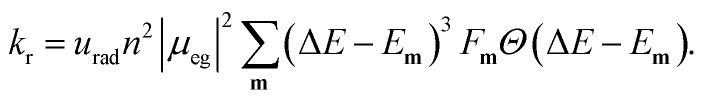




Here, Δ*E* is the adiabatic excitation energy, *E*_***m***_ the energy of the final vibrational state ***m*** (without zero-point energy), *F*_***m***_ = | = |〈0′|0′|***m***〉||^2^ denotes the Franck–Condon factors and the Heaviside function *Θ*(⋅) ensures that the energy difference between the initial state and final state is positive, so that a photon can be emitted. The factor *u*_rad_ = 2.142 × 10^10^ s^–1^ converts the rates from atomic units to s^–1^. For details about the prefactor and how the sum over all vibrational states is performed efficiently, see the ESI.‡

### Nonradiative rates

For the harmonic part of the internal conversion rate we use a variant of the so-called energy gap law.^61^ The detailed derivation from Fermi's Golden rule is given in the ESI‡ and we limit us here to stating the final equation,
6

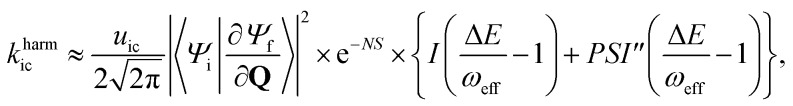

with
7

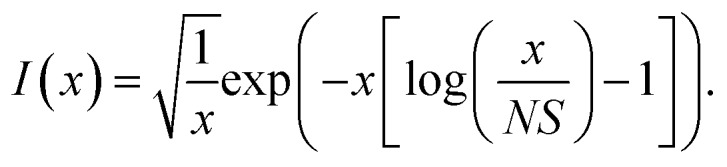




The factor *u*_ic_ = 2π*E*_h_/*ℏ* = 2.598 × 10^17^ s^–1^ converts the rate to s^–1^ if all other quantities are given in atomic units, where *E*_h_ is the Hartree energy. *N* = 168 is the number of vibrational modes denoted by **Q**, *P* is the number of promoting modes, *i.e.*, those modes *p* carrying a large component of the non-adiabatic coupling vector 
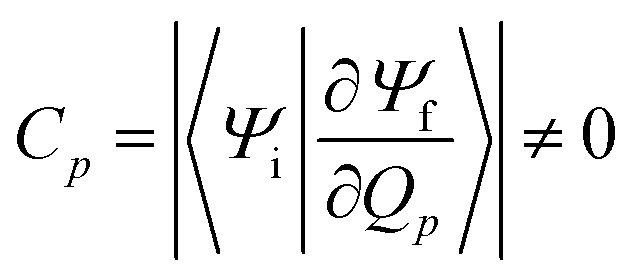
, 
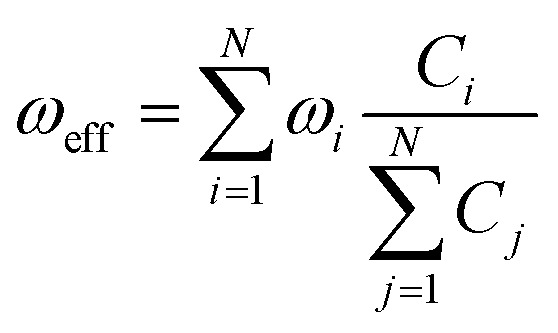
 is the effective vibrational mode, *S* the average Huang–Rhys factor and *I*′′(*x*) denotes the second derivative of *I*(*x*). Averaging over the modes, so that the vibrational structure is described by a single effective mode *ω*_eff_, is a drastic simplification that introduces some arbitrariness. The advantage is that it provides us with a closed expression for the internal conversion rate that brings out the dependence on the adiabatic excitation energy Δ*E*.

The second pathway with rate constant *k*CIic is the relaxation through conical intersections (CIs), which may lead back to the reactant well or to the formation of a photoproduct. Assuming that reaching the CI always leads to an internal conversion from the excited state to the electronic ground state, then the rate for depletion of S_1_ is only dependent on the energetics of the barrier and the vibrational energy of the molecule.

This rate is calculated by Kramers' barrier-crossing theory,^62^ where the decay process starts from an equilibrated initial state characterized by a well frequency *ω*_*a*_ and proceeds over a barrier of height *E*_A_ and frequency *ω*_*b*_ in the presence of a friction constant *γ*,
8

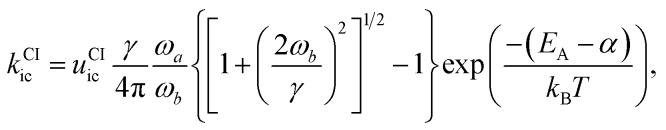

where *α* is a global adjustable parameter as discussed in section 6 of the ESI,‡ *u*CIic = *E*_h_/*ℏ* = 4.134 × 10^16^ s^–1^ converts the rate to s^–1^ if all other quantities are given in atomic units and *ω*_*b*_ is the frequency of the mode with negative force constant at the lowest transition state leading to a conical intersection. Since Kramers' model is essentially one-dimensional, we had to select the corresponding mode a with frequency *ω*_*a*_ at the Franck–Condon point which is most similar to the mode b at the transition state. The detailed calculation of the friction constants *γ* is given in the ESI.‡ The approach based on eqn (5), (6) and (8) is one of the simplest models that can be used to rationalize the photoluminescence quantum yields and can predict qualitative trends based on temperature and solvent environment including the viscosity and solvatochromic shifts.

### Barrier for nonradiative relaxation

**Fig. 3:**
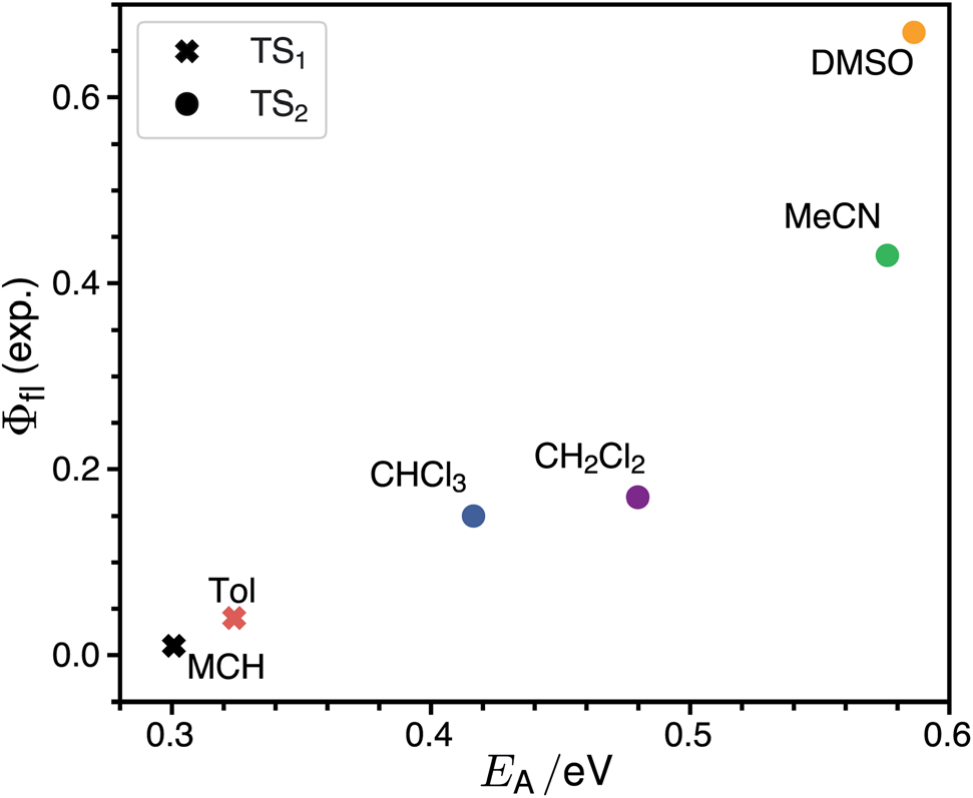
Correlation between the activation energy, *E*_A_, in the S_1_ state from the Franck–Condon minimum to the transition state and the experimental fluorescence quantum yield. For each solvent the transition state lowest in energy is selected.

The rate for internal conversion through the conical intersection is dependent on the energetics of the path to the CI from the Franck–Condon minimum. It has been shown by Maeda^25^ and coworkers that if the internal conversion is the dominating relaxation pathway to the ground state, then the trend in the fluorescence quantum yields can be predicted very well by correlating these barrier heights to the experimentally obtained quantum yield. We observe an almost linear dependence between these quantities, suggesting that this is indeed the dominant relaxation pathway (Fig. 3). Since both the rotation of the double bond which is connected directly to the benzothiazolidene group, and the rotation about the next double bond lead to a conical intersection, there are also two different transition states (denoted as TS_1_ and TS_2_ in Fig. 3).

**Fig. 4:**
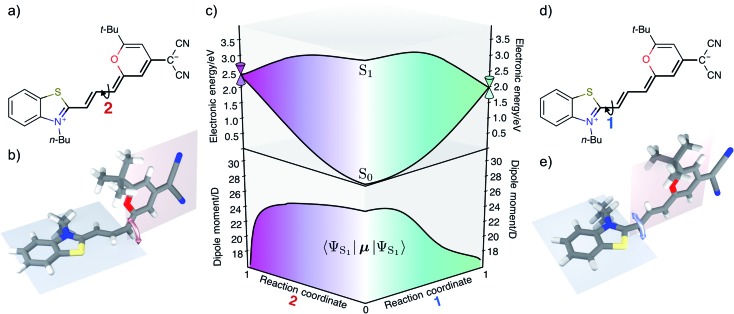
(a and d) In the zwitterionic resonance structure rotation around the former double bonds in the polymethine chain becomes possible leading to conical intersection 1 (e) or 2 (b). (c) CASSCF energies (top) and excited state dipole moments (bottom) in the gas phase along linearly interpolated geometries (in internal coordinates) between the Franck–Condon minimum and the two minimum energy conical intersection as indicated on the left and right hand sides.

We have validated the whole reaction path at the CASSCF level, as linear-response TD-DFT has inherent problems in describing the S_0_/S_1_ conical intersection seam correctly.^63^ In Fig. 4 we illustrate the geometries at the conical intersection together with the energies and permanent dipole moments, from the Franck–Condon point to both conical intersections, of the S_1_ state in the frame of CASSCF. In the gas phase the permanent dipole moment of the excited state decreases from ≈27 D at the Franck–Condon point to less than 16 D at the conical intersections. At the Franck–Condon point we can compare the change in the permanent dipole, Δ*μ*, between the ground and excited state with experiment, which is with a value of 10.5 D in good accordance to the experimental value of Δ*μ* = 9.5 ± 1.1 D (as obtained from the EOA measurements shown in Fig. S6‡).

### Fluorescence quantum yield

The calculated non-radiative decay rates are shown in Table 1 together with the simulated radiative rates and the resulting fluorescence quantum yields. The calculated adiabatic excitation energy Δ*E* decreases from 2.69 eV in the least polar solvent MCH to 2.34 eV in the most polar solvent DMSO. The harmonic radiative rates remain more or less constant for all solvents, since *μ*_eg_ increases from 14.0 D in MCH to 15.5 D in DMSO, so that the opposite trends of decreasing optical gap and increasing transition dipole moments cancel each other. Compared to experiment the radiative rate is overestimated by a factor of ≈3–6. This is partly due to the overestimation of the transition dipole moment by TDDFT relative to experiment (15.5 *vs.* 11.1 D in DMSO). The harmonic rate for internal conversion increases with the positive solvatochromic shift as expected from the energy gap law. This effect is enhanced by the increase of the electronic non-adiabatic coupling *C* from 0.60 eV in MCH to 0.99 eV in DMSO. The trend of the harmonic part of the IC rate alone is opposite to the experimental observation that the non-radiative rates decrease with decreasing emission energy: the experimental *k*_nr_ attains the largest value of 71 × 10^8^ s^–1^ for MCH and the lowest value of 1.4 × 10^8^ s^–1^ for DMSO.

The inclusion of the rate from Kramers' theory reverses the theoretical trend. *k*CIic has its largest value of ≈3 × 10^12^ s^–1^ for MCH and decreases with increasing polarity to 0.22 × 10^8^ s^–1^ in DMSO. In MCH the theoretical rate is dominated by *k*CIic, while in DMSO it is determined mostly by *k*harmic. In the other solvents the dominant non-radiative decay channel changes gradually from being non-reactive (transition from the Franck–Condon point to the S_0_ minimum) to being reactive (decay through a conical intersection). The solvent also determines which of the two conical intersections is accessed. In MCH and toluene the lowest transition state leads to conical intersection 1, while in the other more polar solvents the transition state to the conical intersection 2 is lower. Therefore one can expect *cis*–*trans* isomerization around the first double bond in solvents of low polarity and around the second double bond in solvents of high polarity. The branching ratios for returning to the ground state or *cis*–*trans* isomerization at the conical intersections could be very sensitive to the shape of the intersection seam. Making statements about the isomerization yield is therefore beyond the scope of our theoretical model.

Due to the fitting of the global parameter to *α* = 0.192 eV the agreement between experimental and theoretical fluorescence quantum yields is quite good, although the theoretical rates are off by multiples. In MCH the calculated fluorescence quantum yield is 0, because the barrier to one or the other conical intersection can be overcome easily, whereas this channel is inhibited in DMSO, thereby raising the fluorescence quantum yield to ≈60%. The fluorescence quantum yield does not go to 100% since the rate constant of the non-radiative decay from the Franck–Condon minimum to the ground state increases with the solvatochromic shift.

The single most important factor for explaining the trends in the fluorescence quantum yields is the activation energy. To elucidate how the height of the barrier to the conical intersections is affected by the solvent we characterize the polarity of the ground and excited state by constrained density functional theory followed by configuration interaction (CDFT-CI).

### Qualitative electronic structure analysis

**Fig. 5:**
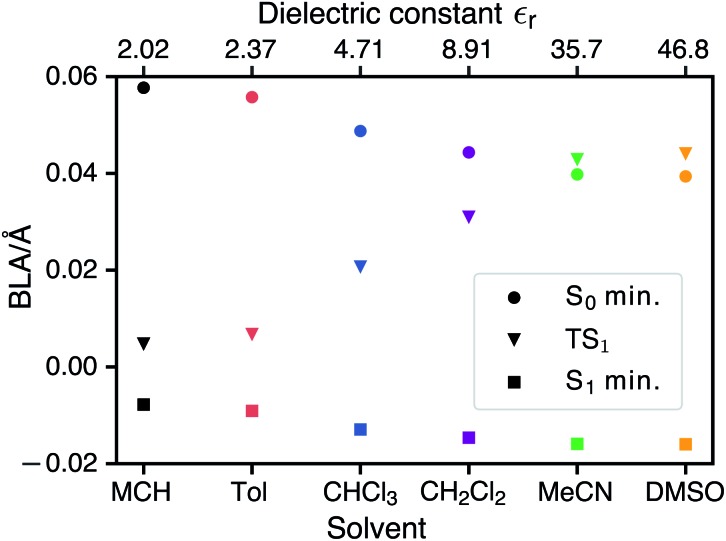
Bond length alternation of the polymethin chain of merocyanine **DCBT** at the ground-state minimum, the Franck–Condon S_1_ minimum and the transition state between this minimum and the conical intersection 1.

CDFT-CI calculations in the gas phase put the weight of the zwitterionic configuration in the ground state at *c*^2^ = 0.267 for the Franck–Condon geometry which is in accordance with an experimental value of *c*^2^ = 0.27 ± 0.02. The weight *c*^2^ is approximately correlated with the pattern of bond length alternation (BLA), which is defined as the average difference in length of single and double bonds which are part of the polymethine chain (in the neutral resonance structure **DA**, see Fig. 1). When *c*^2^ changes from 0 to 1 the double bonds in the polymethine turn into single bonds and *vice versa*. At the same time the BLA switches from positive to negative. Therefore the variations of the bond lengths are a sign of changes in the electronic character of the wavefunction. Fig. 5 shows that the average length of the double bonds increases only slightly at the expense of the single bonds as the dielectric constant of the solvent increases from *ε*_r_ = 2.02 (MCH) to *ε*_r_ = 46.83 (DMSO). The calculated values at the TDDFT/IEFPCM level for the S_0_ minimum (ranging from ≈0.06 Å for MCH to ≈0.04 Å for DMSO) agree very well with the experimental BLA value of 0.044 Å obtained from the crystal structure analysis. The ground state remains dominated by the neutral resonance structure and the excited state by the zwitterionic one for all solvents. However, we observe that the character of the wavefunction at the transition state has a strong dependence on the solvent. The BLA of almost zero in MCH for the TS and the S_1_ minimum indicates that the electronic character is very similar at these geometries and this can be called an early transition state.^28^ As the polarity of the solvent increases, the character of the transition state approaches that of the ground state. Thus, in solvents such as MeCN or DMSO, we are dealing with late transition states. The calculated barrier and experimentally observed trends in the relaxation rates agree well with the qualitative analysis based on the Hammond–Leffler postulate.

### Excited-state solvation

**Fig. 6:**
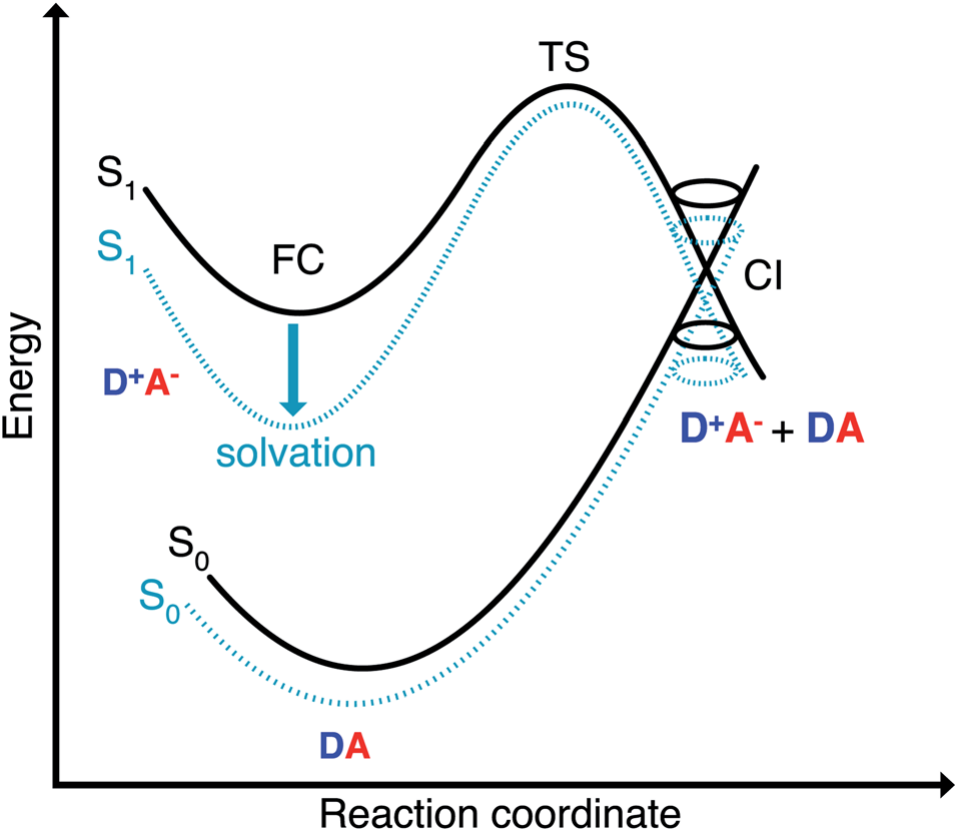
Energetics of thermally activated internal conversion through conical intersection for polyene-type merocyanines (with positive solvatochromism). The different stationary points in the ground and excited state are affected differently by the solvent depending on their polarity.

The Franck–Condon point on S_1_ has the largest excited state dipole moment and its energy is therefore lowered most by a polar solvent (Fig. 6). Compared to this, the ground state with its small dipole moment is lowered much less by the same solvent. Therefore the optical gap decreases as the polarity of the solvent increases. This explains the positive solvatochromic shift observed in the absorption and emission spectrum. The transition state and the conical intersections, which have dipole moments in between the two extremes, are lowered less in energy by the polar solvent than the Franck–Condon point. Therefore the barrier to a conical intersection grows with increasing dielectric constant of the solvent. For the most polar solvents, MeCN and DMSO, the barrier becomes so high that the decay channel through a conical intersection is hardly accessible at room temperature resulting in the observed high fluorescence quantum yields for the most polar solvents. Furthermore, we provide a simple model based on CDFT-CI calculations and Onsager model that can explain why the polarity of the solvent affects different points along the reaction coordinate differently in the ESI.‡

To test our hypothesis we conducted transient absorption spectroscopy which provides evidence for the presence of a decay channel through a conical intersection that can be turned on and off by changing the solvent.

### Transient absorption data

**Fig. 7:**
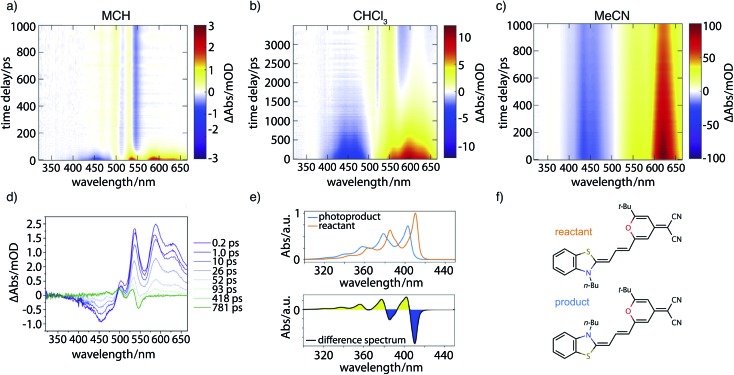
Transient absorption results. Maps are shown for the merocyanine dye **DCBT** in various solvents of increasing polarity using excitation wavelengths *λ*_ex_ tuned to the linear absorption maxima of 0–1 transitions: (a) MCH at *λ*_ex_ = 506 nm, (b) CHCl_3_ at *λ*_ex_ = 518 nm, and (c) MeCN at *λ*_ex_ = 514 nm. (d) Selected transient spectra for the case of MCH. The final shown trace at 781 ps (green) represents the absorption difference between the reactant and an isomerization product. (e) Top: Vibrationally resolved TDDFT absorption spectra in the gas phase of the reactant (red) and isomerization product (blue); bottom: linear difference spectrum between reactant and product that corresponds well to the green curve in d. (f) Corresponding chemical structures for reactant (red) and isomerization product (blue).

Fig. 7 contains the results of the transient absorption experiment. Shown in Fig. 7a–c are transient absorption maps of the merocyanine dye in solvents with increasing polarity, namely methylcyclohexane (Fig. 7a), chloroform (Fig. 7b), and acetonitrile (Fig. 7c). Positive signals (yellow, red) around 500–660 nm correspond to excited-state absorption, negative signals (blue) in the region of 400–500 nm to ground-state bleach. Global analysis leads to decay-associated spectra as presented in the ESI (Fig. S14‡). Obtained lifetimes for all solvents are summarized in Table S6.‡ In the case of the nonpolar solvent MCH (Fig. 7a) global analysis reveals a time constant for the recovery of the ground-state bleach (*τ*_4_ = 36.0 ps) which is in agreement with the measured fluorescence lifetime of less than 120 ps. At long time delays a permanent change in absorption is observed in the range of 450–550 nm that is indicative of the formation of a photoproduct, to be discussed further below.

In the solvent with intermediate polarity (*i.e.*, CHCl_3_, Fig. 7b), the return of the population to the ground state is much slower than in the nonpolar solvent MCH (*τ*_4_ = 834.0 ps) that is consistent with the experimentally determined fluorescence lifetime of 590 ps. We again observe a permanent change in absorption indicating the formation of a photoproduct.

In the most polar solvent MeCN (Fig. 7c) the return to the ground state is even slower than in CHCl_3_ and the time constant for the relaxation back to the ground state (*τ*_5_ = 1.44 ns) is in agreement with the experimentally determined fluorescence lifetime of *τ*_fl_ = Φ_fl_/*k*_r_ = 1.78 ns (see Table 1).

We now further analyze the photoproduct observed at long times in MCH and CHCl_3_, shown exemplarily for the case of MCH in the transient spectrum at 781 ps (Fig. 7d, green curve). We observe characteristic positive and negative features corresponding, respectively, to the rise of product absorption and the permanent bleach of reactant. To identify the photoproduct, we calculated vibrationally resolved TDDFT absorption spectra (Fig. 7e, top) for the reactant (red) and a tentative photoproduct (blue) following *cis*–*trans* isomerization around the first double bond in the polymethine chain, with chemical structures displayed in Fig. 7f. The photoproduct is connected to the initially excited Franck–Condon region by a path over the transition state and a conical intersection at a twisted double bond. The geometry of the isomer is sterically hindered and less stable than the reactant by Δ*G* = 19.96 kJ mol^–1^. The vibrational substructure can be assigned to a progression of the C–C stretch vibration in the polymethine bridge. Since the spectrum of the photoproduct is blue shifted by approximately half the vibrational frequency, the resulting difference spectrum (Fig. 7e, bottom) consists of alternating troughs and peaks. It agrees very well with the experimental difference spectrum at long time delay (Fig. 7d, green).

In summary, the experimental transient absorption data confirm the observed trend of increasing lifetimes with increasing solvent polarity. Furthermore, a permanent change in absorption indicating the formation of a photoproduct can be observed in case of MCH and CHCl_3_. In MeCN such a change was not seen within the maximum delay time of 1 ns. This is in line with the proposed increase of the barrier to the conical intersection with increasing solvent polarity.

### Temperature dependence of fluorescence quantum yields

Measurements of fluorescence quantum yields at lower temperatures (*T* ≈ 250 K and 200 K) provide additional evidence for our theoretical model. Thus, they are in accord with the temperature dependence implied in eqn (3) due to *k*CIic ∝ e^–*E*_A_/(*k*_B_*T*)^ (shown in Fig. S15 in the ESI‡). In all solvents for which experimental data are available over a larger temperature range (limitations are imposed by the melting point of the solvent and dye aggregation at lower temperatures in MCH), the fluorescence quantum yield increases at lower temperatures. Importantly, also the inflection point, where the fluorescence quantum yield increases most, moves to higher temperatures as the polarity of the solvent increases.

## 4 Conclusion

We have synthesized a new dipolar merocyanine dye and measured its fluorescence quantum yield as a function of the solvent polarity and temperature. A positive solvatochromic shift is accompanied by an increase in the fluorescence lifetime which is at odds with the predictions of the energy gap law. An additional decay channel through conical intersections was found by extensive quantum chemical exploration of the potential energy surfaces of the solvated merocyanine and resolved this apparent contradiction. We have presented theoretical and experimental evidence for the presence of conical intersections that are thermally activated and lead to a non-radiative decay involving either return to the reactant minimum or *cis*–*trans* isomerization. The temperature dependence of the non-radiative decay rates proves the existence of a barrier. The activation energy increases with solvent polarity which is explained by the better stabilization of the very dipolar excited state by the polar solvents as illustrated in Fig. 6.

These results are not only of importance for our fundamental understanding of the photophysics of donor–acceptor dyes. Merocyanines like **DCBT** may also open up interesting opportunities as fluorescent probes in biomolecular studies because both the increase of the barrier in the polar solvent water as well as the inhibition of the torsional decay channel by coordination of the dye to proteins or RNA/DNA macromolecules are expected to enable high fluorescence quantum yields.^64^–^66^

## Conflicts of interest

There are no conflicts to declare.

## Supplementary Material

Supplementary information

Crystal structure data
