# Supplementary material for: The origin of the solvent dependence of fluorescence quantum yields in dipolar merocyanine dyes
Source: Chem Sci. 2019 Dec 4;10(48):11013–22. doi: 10.1039/c9sc05012d (PMC7069518; doi:10.1039/c9sc05012d)
Supplement: Supplementary file 1 [file SC-010-C9SC05012D-s001.pdf]

Electronic Supplementary Material (ESI) for Chemical Science.
This journal is © The Royal Society of Chemistry 2019

Electronic Supplementary Material

The Origin of the Solvent Dependence of Fluorescence Quantum Yields in Dipolar Merocyanine Dyes

Joscha Hoche[a], Alexander Schulz[bc], Lysanne Monika Dietrich[ac], Alexander Humeniuk[a], Matthias Stolte[bc], David Schmidt[bc], Tobias Brixner[ac], Frank Würthner[bc], Roland Mitrić[ac]

[a] *Institut für Physikalische und Theoretische Chemie, Julius-Maximilians-Universität Würzburg, D-97074, Würzburg, Germany E-mail: tobias.brixner@uni-wuerzburg.de, roland.mitric@uni-wuerzburg.de*
[b] *Institut für Organische Chemie, Julius-Maximilians-Universität Würzburg, D-97074, Würzburg, Germany E-mail: wuerthner@uni-wuerzburg.de*
[c] *Center for Nanosystems Chemistry & Bavarian Polymer Institute (BPI), Julius-Maximilians-Universität Würzburg, D-97074, Würzburg, Germany*

Contents

1 Experimental Details **S3**
- 1.1 Synthetic Details S3
- 1.2 UV/Vis and Fluorescence Spectroscopy S3
- 1.3 Temperature-dependent Spectroscopy S3
- 1.4 Electrooptical Absorption Measurements. S4
- 1.5 Crystallographic Analysis S4
- 1.6 Broadband Ultrafast Transient Absorption S4
- 1.7 Computational Details S5

2 Synthesis and Characterization **S6**
- 2.1 3-Butyl-2-[2-(methylphenylamino)ethenyl]benzothiazolium iodide (3) S6
- 2.2 4-(Dicyanomethylene)-2-*tert*-butyl-6-[3-(3-butyl-benzothiazol-2-ylidene)1-propenyl]-4H-pyran (DCBT merocyanine) S7
- 2.3 Structural Elucidation S10

3 Crystallographic Analysis **S10**
- 3.1 Crystallographic data S10

4 Fluorescence decay and optical properties **S14**

5 Rates based on harmonic approximation and Fermi's Golden Rule **S16**
- 5.1 Fluorescence Rates S16
- 5.2 Internal Conversion Rates - Time-Independent S17
- 5.3 Internal Conversion Rates - Time-Dependent S19
  - 5.3.1 Energy gap law - saddle point integration S22
- 5.4 Notes on the saddle point approximation S25
- 5.5 Nonadiabatic Couplings S28

6 Rates based on Kramers's Theory **S30**
- 6.1 Calculation of Friction Coefficients S30

7 Onsager Solvation Model **S31**

8 Transient Absorption Spectroscopy **S33**

9 Temperature Dependence of Quantum Yields **S34**

1 Experimental Details

1.1 Synthetic Details

All reagents were purchased from commercial sources and used without further purification, unless otherwise stated. *N*-Methylaniline was distilled under inert conditions and stored in a Schlenk flask. Column chromatography was performed with commercial glass columns using silica gel 60M (particle size 0.040063 mm) as stationary phase, whereas flash column chromatography was carried out with a PuriFlash 420 system of Interchim using commercially available silica gel columns (PF-30SIHP-F0025). Melting points were determined using an Olympus BX41 polarisation microscope and are not corrected. ^{1}H und ^{13}C NMR spectra were measured on a Bruker Avance III HD 400 MHz spectrometer and calibrated to the residual protic solvent signal.

1.2 UV/Vis and Fluorescence Spectroscopy

Steady-state absorption spectra were recorded using a V770 UV-Vis spectrometer (JASCO Inc., Japan) and emission spectra were measured with a FLS980-D2D2-ST (Edinburgh Instruments Ltd., UK) fluorescence spectrometer and corrected against the photomultiplier sensitivity and the lamp intensity. All spectra were recorded at 298 K, if not stated otherwise, and the temperature was controlled by a sample holder with Peltier element. The fluorescence quantum yields (Φ_{fl}) were determined as average value of four different excitation wavelengths relative using *N,N'*-(2,6-di-iso-propylphenyl)-1,6,7,12-tetraphenoxyperylene-3,4:9,10-tetracarboxylic acid bisimide ($\Phi_{\text{fl}} = 0.96$ in chloroform1) and *N,N'*-bis(2,6-di-iso-propylphenyl)-perylene-3,4:9,10-tetracarboxylic acid bisimide ($\Phi_{\text{fl}} = 1.00$ in chloroform) as standards under highly diluted conditions (OD ≤ 0.05) and magic angle conditions (54.7°). The fluorescence lifetimes were determined by Time Correlated Single Photon Counting (TCSPC) using an EPL picosecond pulsed diode laser ($\lambda_{\text{fl}} = 505.8$ nm) with a pulse width of 141.7 ps with an FLS980-D2D2-ST spectrometer (Edinburgh Instruments Ltd., UK) under magic angle conditions (54.7°). The fitting of the data was carried out using the Tail-Fit-routine supplied by Edinburgh Instruments Ltd., Inc.

1.3 Temperature-dependent Spectroscopy

Temperature-dependent absorption and emission spectra were recorded with a JASCO V770 and FLS980 spectrometer, respectively using a Cold Finger Dewar by PTI. A highly diluted sample (OD ≤ 0.05) was placed in a commercial NMR tube and cooled using *o*-xylene/dry ice (-25 °C) or acetone/dry ice (-78 °C) bath. The quantum yield at lower temperature

was determined by choosing an excitation wavelength, which showed no changes of the optical density upon cooling and comparing the integral of the resulting fluorescence spectra at different temperatures. Cooling with acetone/dry ice could only be used for dichloromethane as **DCBT** aggregated in methylcyclohexane and chloroform freezes at this temperature.

1.4 Electrooptical Absorption Measurements.

Electrooptical Absorption Measurements (EOAM) were carried out using a self-constructed spectrometer in methylcyclohexane at 298 K and evaluated according to literature known procedure[1,2].

1.5 Crystallographic Analysis

Single crystals of **DCBT** suitable for X-ray crystallographic analysis were obtained by slow evaporation of hexane into a concentrated dichloromethane solution. Single crystal X-ray data were collected at 100 K on a Bruker D8 Discover Diffractometer with a LynxEye-1D-Detector and multi-layered mirror monochromated CuK_α radiation. The structures were solved using direct methods expanded with Fourier techniques and refined with the Shelx software package. All non-hydrogen atoms were refined anisotropically. Hydrogen atoms were included in the structure factor calculation on geometrically idealized positions. Crystallographic data have been deposited with the Cambridge Crystallographic Data Centre as supplementary publication no. CCDC 1957268.
These data can be obtained free of charge from The Cambrige Crystallographic Data Centre via `www.ccdc.cam.ac.uk/data_request/cif`.

1.6 Broadband Ultrafast Transient Absorption

We employed ultrafast transient absorption in the ultraviolet/visible (UV/Vis) region as described in detail elsewhere[3–5]. A commercial Ti:sapphire oscillator (Solstice, Spectra-Physics) provided pulses with a duration of 120 fs and a central wavelength of 800 nm at a repetition rate of 1 kHz. The $\approx$ 50 fs pump pulses (with varying central excitation wavelengths of λ_{ex} = 506 nm, 514 nm, and 518 nm) were derived from the second-harmonic output of a commercial nonlinear optical parametric amplifier (TOPAS White, Light Conversion). The broadband UV/Vis probe pulses were generated by focusing a small fraction of the residual 800 nm light into a linearly moving CaF_2 disk, generating a white-light continuum from 350 nm to 650 nm. For the transient absorption experiments, pump and probe pulses were focused individually and non-collinearly to achieve spatial overlap in a flow cell with a sample thickness of 200 μm, which at the chosen sample concentration led to optical densities of 0.3–0.5 for the various solvents. The relative polarizations of the pump and the probe beams were

set to the magic angle of 54.7° by turning the pump polarization with achromatic waveplates. The pumpprobe delay was varied by delaying the probe beam with a mechanical translation stage (M-IMS600, Newport) up to 1.0 ns in methylcyclohexane and acetonitrile and up to 3.5 ns in chloroform. Every second pump pulse was blocked by a chopper driven at 500 Hz. After passing the sample, the UV/Vis probe pulses were detected shot-to-shot using a spectrometer (Acton SP2500i, Princeton Instruments) with an attached CCD camera (Pixis 2K, Princeton Instruments). The transient UV/Vis data were evaluated via target analysis[6] with the software package Glotaran[7] based on the R-package TIMP[8].

1.7 Computational Details

For the simulation of vibrationally resolved absorption and fluorescence spectra of the studied system, the structure has been first optimized in the framework of density functional theory (DFT), using the long-range corrected ωB97XD functional[9] and the def2-TZVP basis set[?] as implemented in the Gaussian 16 quantum chemical software package[10]. The excited state properties have been calculated in the frame of time-dependent density functional theory (TDDFT) employing the same functional and basis set. The solvent effects are treated in an implicit way via the polarizable continuum model using the integral equation formalism variant (IEFPCM)[11–15]. When excited state properties are calculated, the reaction field of the solvent is adjusted to the electronic density of the excited state (equilibrium solvation). The vibrationally resolved absorption spectra for the lowest excited state and the vibrationally resolved emission spectra from the lowest singlet excited state were simulated within the Franck-Condon approximation[16]. For this purpose the geometries of the ground and excited electronic states have been optimized and the harmonic vibrational analysis has been performed. In the spectral simulations, the adiabatic hessian (AH) model, which takes the the Duschinsky transformation[17] into account, has been used.
The S_0/S_1-MECI geometry of the merocyanine dye was searched using the projected gradient method by Bearpark et. al[18] at the level of the CASSCF[19–22] methodology as implemented in the Brilliantly Advanced General Electronic-structure Library (BAGEL) by Shiozaki and coworkers[23]. The four higherlying occupied π orbitals and the four lower virtual ones, using 8 π electrons, were included in the active space of the CASSCF calculations. Starting orbitals have been generated by performing HF calculations and have been optimized in a state-averaged manner with equal weights for the S_0 and S_1 states. Starting from the FC point we explored the minimum energy conical intersections (MECI's) between the S_0 and the S_1 at the CASSCF level in gas phase. The obtained geometries show rotations of either one of the double bonds connecting the benzothiazole group and the polymethine chain by about 90 degree. For the calculation of thermodynamic properties, we optimized the transition state, which connects the FC minimum and the MECI geometry, in the first excited state

at the linear-response TDDFT level by using the ωB97XD functional and the def2-TZVP basis set. The Gibbs free energy of the S_1 minimum and the transition state geometry were calculated at 298.15 K and 1 atm.
The constrained density functional theory[24,25] calculations at the B3LYP/def2-SVP level in combination with configuration interaction were done using the Q-Chem program suite[26].

2 Synthesis and Characterization

The target compound was synthesized according to the literature known procedure depicted in scheme 1[27]. Starting compounds **1**[28] and **4**[29,30] were prepared according to literature.

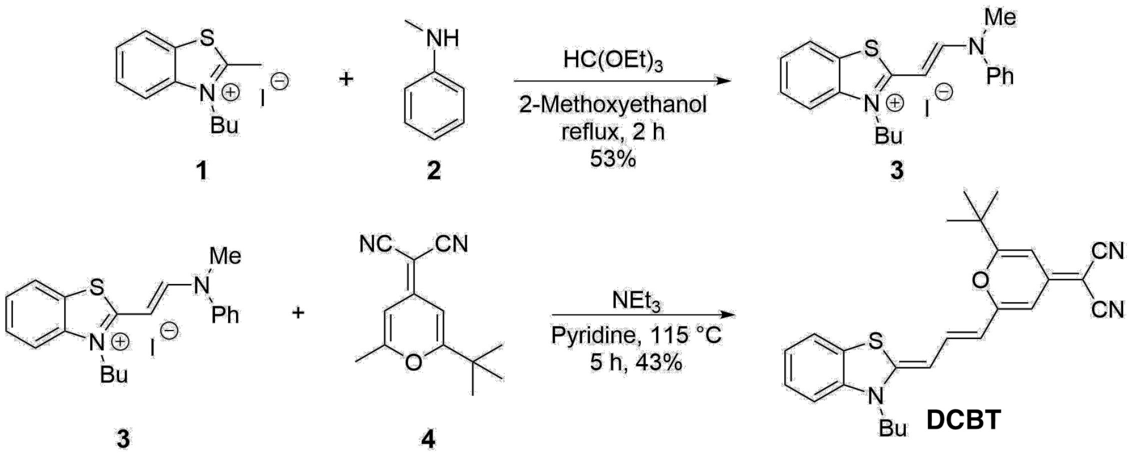

Scheme 1: Synthesis of the target merocyanine **DCBT**.

2.1 3-Butyl-2-[2-(methylphenylamino)ethenyl]benzothiazolium iodide (3)

Compound **1** (1.97 g, 5.91 mmol), *N*-methylaniline (1.92 mL, 0.99 g/mL, 1.90 g, 17.7 mmol) and triethylorthoformate (2.69 mL, 0.90 g/mL, 2.41 g, 16.2 mmol) were dissolved in 2-methoxyethanol and heated under reflux for 2 h. The obtained solution was cooled down to room temperature and treated with diethylether (400 mL). The precipitate was collected by filtration, washed with acetone (30 mL) and diethylether (400 mL). The solid was subsequently dissolved in methanol (20 mL) and precipitated again with diethylether (450 mL) to give **3** (1.42 g, 53%) as a purple solid. The compound was used without further purification. ^{1}H NMR (400 MHz, CD_2Cl_2): δ = 8.03 (d, J = 12.6 Hz, 1H), 7.89-7.34 (m, 9H), 6.37 (d, J = 12.6 Hz, 1H), 4.68 (t, J = 7.4 Hz, 2H), 3.84-3.81 (m, 3H), 1.96-1.85 (m, 2H), 1.64-1.54 (m, 2H), 1.03 (t, J = 7.2 Hz, 3H) ppm. ^{13}C NMR (101 MHz, CD_2Cl_2): δ = 170.7, 153.3, 146.0, 141.4, 130.4, 128.9, 128.0, 126.9, 123.4, 122.2, 114.5, 99.8, 90.1, 48.7, 41.1, 30.3, 20.5, 14.0 ppm. HRMS (ESI-TOF, positive, MeCN/$CHCl_3$): m/z calculated for $C_{20}H_{23}N_2S^+$ $[M-I]^+$: 323.1577, found: 323.1576. Melting point: 70-73 °C.

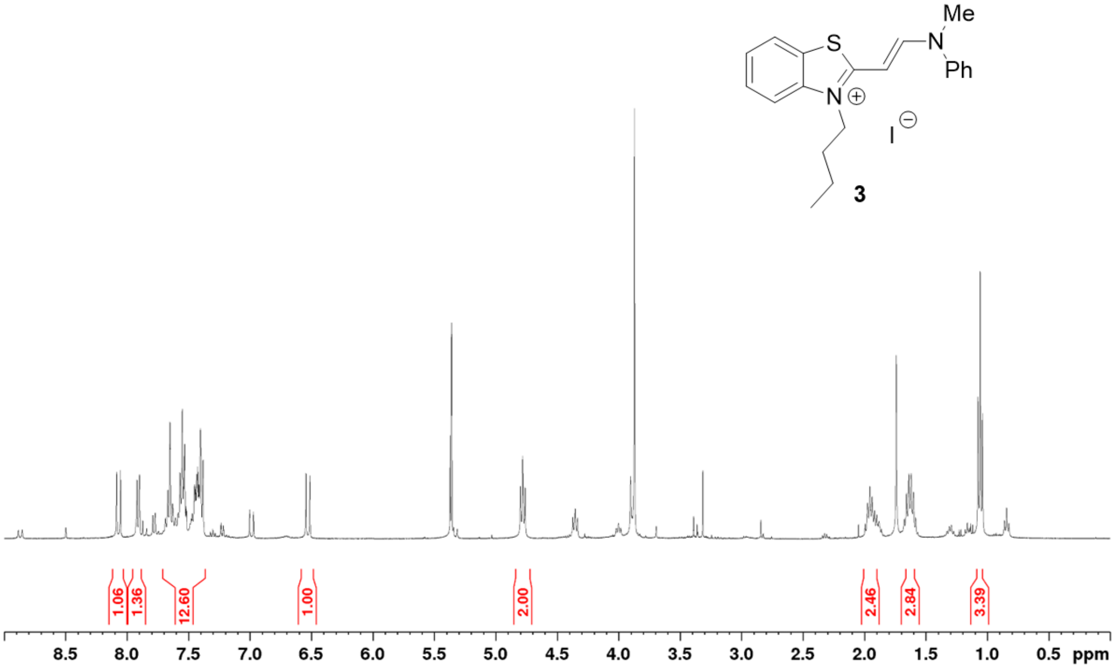

Fig. S1: ^{1}H NMR spectrum (400 MHz) of compund **3** in CD_2Cl_2 at 298 K

2.2 4-(Dicyanomethylene)-2-*tert*-butyl-6-[3-(3-butyl-benzothiazol-2-ylidene)1-propenyl]-4H-pyran (DCBT merocyanine)

A suspension of compound **3** (180 mg, 400 μmol) and compound **4** (64.3 mg, 300 μmol) in a mixture of pyridine (1 mL) and triethylamine (0.2 mL) was stirred at 115 °C for 5 h. After cooling down to room temperature, the solvent was removed under reduced pressure and the crude product was purified by column chromatography (silica, cyclohexane/CH_2Cl_2 1:2), followed by flash chromatography (silica, cyclohexane to cyclohexane/CH_2Cl_2 1:1) to afford pure **DCBT** (55.8 mg, 43%) as a dark purple solid. ^{1}H NMR (400 MHz, CD_2Cl_2): δ = 7.43-7.39 (m, 2H), 7.31-7.27 (m, 1H), 7.09-7.05 (m, 1H), 6.98-6.96 (m, 1H), 6.41 (d, J = 2.0 Hz, 1H), 6.35 (d, J = 2.0 Hz, 1H), 5.86 (d, J = 14.3 Hz, 1H), 5.57 (d, J = 11.9 Hz, 1H), 3.86 (t, J = 7.7 Hz, 2H), 1.77-1.69 (m, 2H), 1.51-1.43 (m, 2H), 1.37 (s, 9H), 1.00 (t, J = 7.3 Hz, 3H) ppm. ^{13}C NMR (101 MHz, CD_2Cl_2): δ = 171.5, 161.9, 156.6, 155.7, 142.6, 139.4, 127.2, 124.6, 122.7, 121.9, 116.98, 116.96, 110.3, 108.5, 102.7, 101.9, 91.6, 54.5, 45.3, 36.7, 29.1, 28.3, 20.6, 14.0 ppm. HRMS: (ESI-TOF, positive, MeCN/$CHCl_3$): m/z calculated for $C_{26}H_{28}N_3OS^+$ $[M^+H]^+$: 430.1948, found: 430.1940. UV/Vis (CH_2Cl_2, $c = 2.06 \cdot 10^{-5}$ M): $\lambda_{max} = 559$ nm ($\epsilon = 70500$ M^{-1} cm^{-1}). melting point: 244-246 °C.

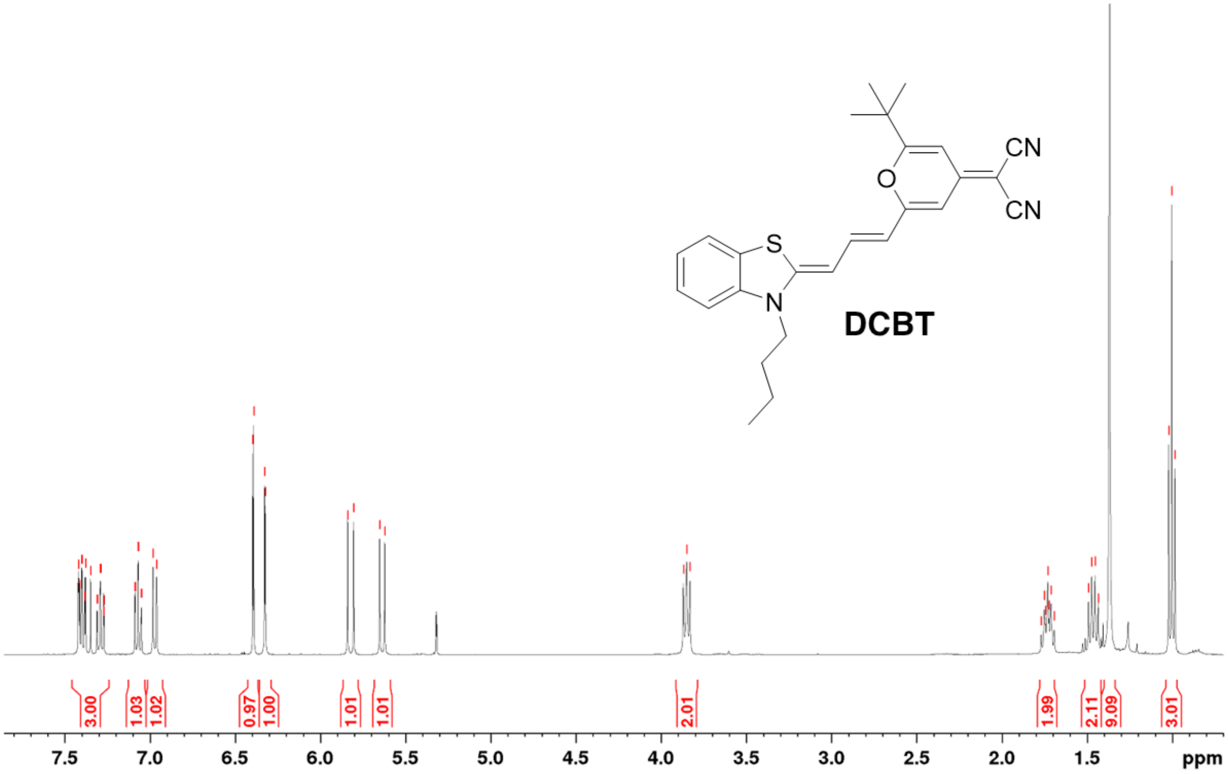

Fig. S2: ^{1}H NMR spectrum (400 MHz) of **DCBT** in CD_2Cl_2 at 298 K

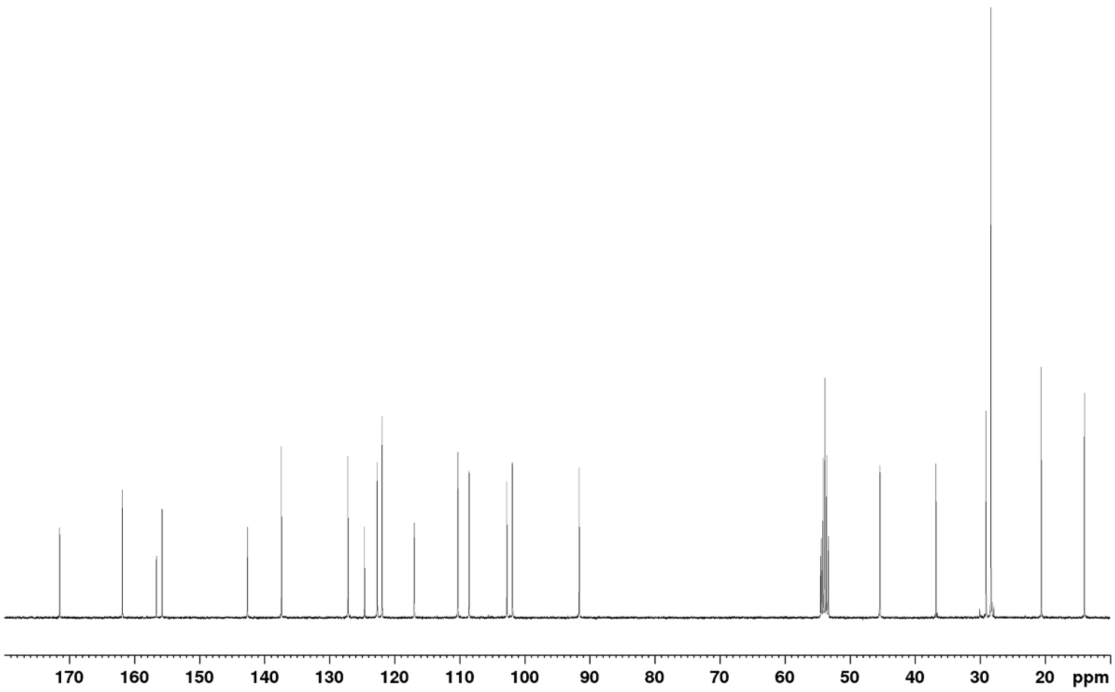

Fig. S3: ^{13}C NMR spectrum (101 MHz) of **DCBT** in CD_2Cl_2 at 298 K

2.3 Structural Elucidation

The proton and carbon signals of **DCBT** were assigned by ROESY, COSY, HSQC and HMBC NMR spectroscopy. From the obtained data the ground state structure in solution could be determined to be the all-trans structure reported for similar chromophores like 4-(Dicyanomethylene)-2-methyl-6-(4-dimethylaminostyryl)-4H-pyran. This could further be proven by an obtained crystal structure of the compound.

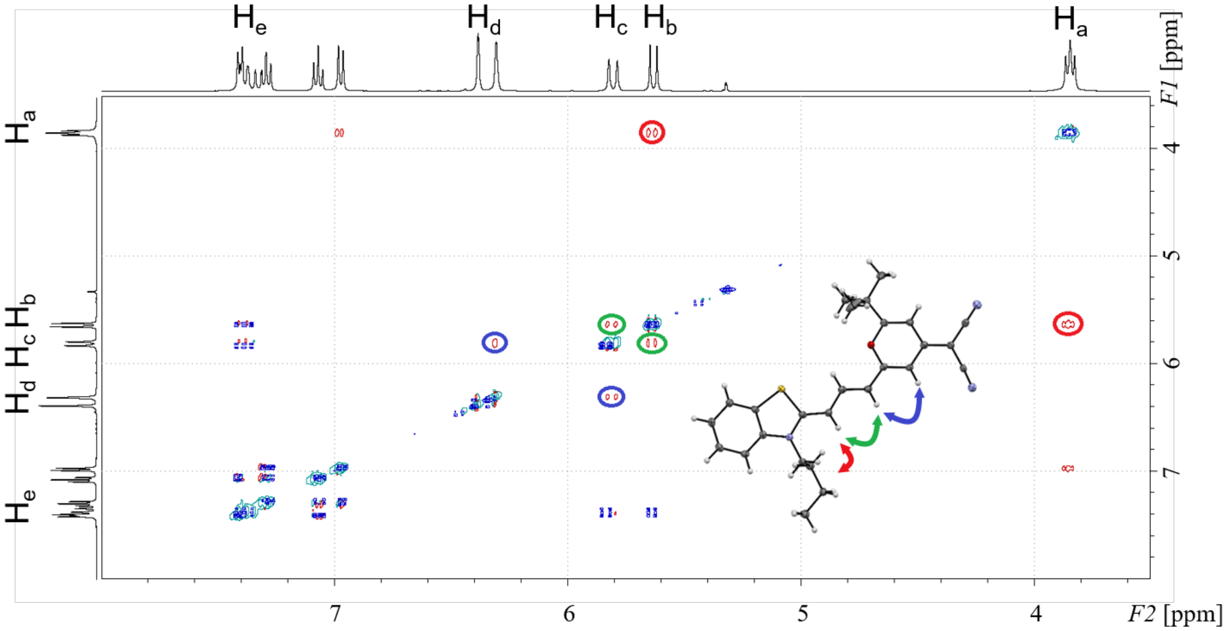

Fig. S4: An Excerpt of the ROESY spectrum (400 MHz) in CD_2Cl_2 at 298 K with the obtained crystal structure of **DCBT**. The observed ^{1}H-^{1}H couplings are marked by colored circles in the spectrum and correlations are indicated with double arrows in the structure. Additionally to the positive (red) and negative (turquoise) ROESY-signals the COSY signals (blue) of the compound are depicted.

3 Crystallographic Analysis

Crystal data for **DCBT**: $C_{26}H_{27}N_3OS$, Mr = 429.56 g mol^{-1}, red needle-shaped crystal, triclinic space group P-1, a = 7.0287(2) Å, b = 11.0372(3) Å, c = 15.2233(5) Å, α = 76.459(1)°, β = 89.714(1)°, γ = 78.323(1)°, V = 1123.28(6) Å^3, Z = 2, ρ_{calcd} = 1.270 g cm^{-3}, μ = 1.451 mm^{-1}, $F(000)$ = 456, T = 100(2) K, $R1$ = 0.037, $wR2$ = 0.095, 4415 independent reflections [$2\Theta \leq 72.26°$] and 284 parameters.

3.1 Crystallographic data

Compound	**DCBT**
Formula	$C_{26}H_{27}N_3OS$
Molecular weight (g·mol^{-1})	429.56
Temperature (K)	100(2)
Wavelength (Å)	1.54178
Crystal system	triklin
Space group	P-1
Unit cell dimensions	
a (Å)	7.0287(2)
b (Å)	11.0372(3)
c (Å)	15.2233(5)
a (°)	76.459(1)
b (°)	89.714(1)
g (°)	78.323(1)
Volume (Å^3)	1123.28(6)
Z	2
Calculated density (Mg·m^{-3})	1.27
Absorption coefficient (mm^{-1})	1.451
F(000)	456
Theta range for data collection	2.99° to 72.26°
Reflections collected	19865
Reflections unique	4415
minimal/maximal Transmission	0.522 / 0.754
Refinement method	Full-matrix least-squares on F2
Data / Parameters / Restrains	4415 / 284 / 0
Goodness-of-fit for F2	1.04
Final R Indices [I >2s(I)]	R1 = 0.037, wR2 = 0.095
R Indices (all data)	R1 = 0.042, wR2 = 0.098
Largest difference peak and hole (e·Å^{-3})	0.28 / 0.31

Table S1: Atomic coordinates (x 10^4) and equivalent isotropic displacement parameters (A^2 x 10^3) for **DCBT**. U(eq) is defined as one third of the trace of the orthogonalized U_{ij} tensor.

	x	y	z	U(eq)
S1	9323(1)	912(1)	7736(1)	17(1)
O1	2173(1)	3054(1)	8300(1)	17(1)
N1	9964(2)	1826(1)	6066(1)	16(1)
N2	3963(2)	6248(1)	5700(1)	26(1)
N3	-6086(2)	5624(1)	8520(1)	28(1)
C1	11622(2)	414(1)	7335(1)	17(1)
C2	13220(2)	435(1)	7814(1)	20(1)
C3	14915(2)	696(1)	7355(1)	21(1)
C4	14994(2)	117(1)	6443(1)	20(1)
C5	13403(2)	731(1)	5962(1)	18(1)
C6	11704(2)	996(1)	6418(1)	16(1)
C7	8498(2)	1897(1)	6665(1)	16(1)
C8	6666(2)	2623(1)	6462(1)	17(1)
C9	5186(2)	2734(1)	7088(1)	17(1)
C10	3374(2)	3452(1)	6831(1)	17(1)
C11	1794(2)	3645(1)	7406(1)	16(1)
C12	4(2)	4368(1)	7107(1)	16(1)
C13	1515(2)	4543(1)	7709(1)	16(1)
C14	1038(2)	3905(1)	8643(1)	18(1)
C15	750(2)	3188(1)	8906(1)	17(1)
C16	9660(2)	2532(1)	5118(1)	17(1)
C17	8640(2)	1864(1)	4548(1)	18(1)
C18	8365(2)	2576(1)	3554(1)	18(1)
C19	10201(2)	2389(1)	3030(1)	22(1)
C20	3356(2)	5282(1)	7412(1)	17(1)
C21	3752(2)	5833(1)	6470(1)	19(1)
C22	4873(2)	5474(1)	8020(1)	20(1)
C23	1479(2)	2453(1)	9852(1)	22(1)
C24	3015(4)	3082(2)	10170(1)	54(1)
C25	2399(3)	1075(2)	9843(1)	32(1)
C26	195(3)	2430(2)	10493(1)	44(1)

Table S2: Anisotropic displacement parameters (10^3 Å^2) for **DCBT**.

	U_{11}	U_{22}	U_{33}	U_{23}	U_{13}	U_{12}
S1	16(1)	18(1)	16(1)	2(1)	3(1)	1(1)
O1	16(1)	16(1)	16(1)	3(1)	1(1)	1(1)
N1	16(1)	17(1)	16(1)	2(1)	3(1)	2(1)
N2	20(1)	32(1)	22(1)	1(1)	1(1)	1(1)
N3	23(1)	32(1)	23(1)	3(1)	5(1)	3(1)
C1	17(1)	16(1)	18(1)	5(1)	3(1)	5(1)
C2	20(1)	19(1)	18(1)	3(1)	1(1)	2(1)
C3	17(1)	21(1)	24(1)	4(1)	0(1)	0(1)
C4	16(1)	21(1)	24(1)	8(1)	5(1)	3(1)
C5	20(1)	18(1)	16(1)	5(1)	3(1)	5(1)
C6	15(1)	14(1)	18(1)	5(1)	1(1)	4(1)
C7	18(1)	14(1)	18(1)	4(1)	3(1)	6(1)
C8	18(1)	15(1)	17(1)	2(1)	1(1)	2(1)
C9	19(1)	14(1)	18(1)	4(1)	2(1)	4(1)
C10	19(1)	15(1)	17(1)	3(1)	2(1)	4(1)
C11	19(1)	12(1)	16(1)	3(1)	1(1)	4(1)
C12	18(1)	14(1)	15(1)	2(1)	1(1)	4(1)
C13	18(1)	12(1)	19(1)	4(1)	2(1)	4(1)
C14	19(1)	16(1)	17(1)	4(1)	4(1)	2(1)
C15	21(1)	14(1)	17(1)	5(1)	4(1)	3(1)
C16	18(1)	15(1)	17(1)	1(1)	3(1)	3(1)
C17	20(1)	14(1)	20(1)	2(1)	1(1)	4(1)
C18	18(1)	16(1)	18(1)	4(1)	0(1)	1(1)
C19	24(1)	22(1)	22(1)	7(1)	4(1)	4(1)
C20	16(1)	16(1)	19(1)	3(1)	2(1)	3(1)
C21	22(1)	19(1)	25(1)	4(1)	3(1)	2(1)
C22	19(1)	17(1)	20(1)	2(1)	1(1)	1(1)
C23	26(1)	19(1)	17(1)	1(1)	1(1)	1(1)
C24	83(2)	45(1)	32(1)	10(1)	30(1)	31(1)
C25	40(1)	24(1)	22(1)	1(1)	5(1)	8(1)
C26	45(1)	50(1)	17(1)	5(1)	10(1)	19(1)

4 Fluorescence decay and optical properties

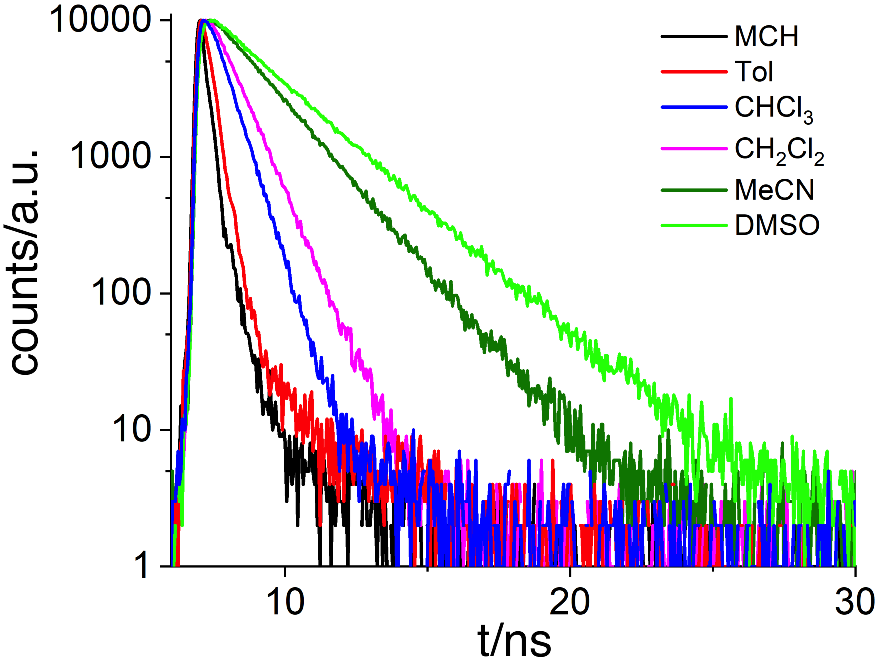

Fig. S5: Fluorescence decay curves of **DCBT** excited with an EPL picosecond pulsed diode laser (λ_{ex} = 505.8 nm) in methylcyclohexane (black, λ_{em} = 546.00 nm), toluene (red, λ_{em} = 579.00 nm), chloroform (blue, λ_{em} = 598.00 nm), dichlormethane (magenta, λ_{em}= 606.00 nm), acetonitrile (dark green, λ_{em} = 623.00 nm) and dimethylsulfoxide (light green, λ_{em} = 637.00 nm) at 298 K.

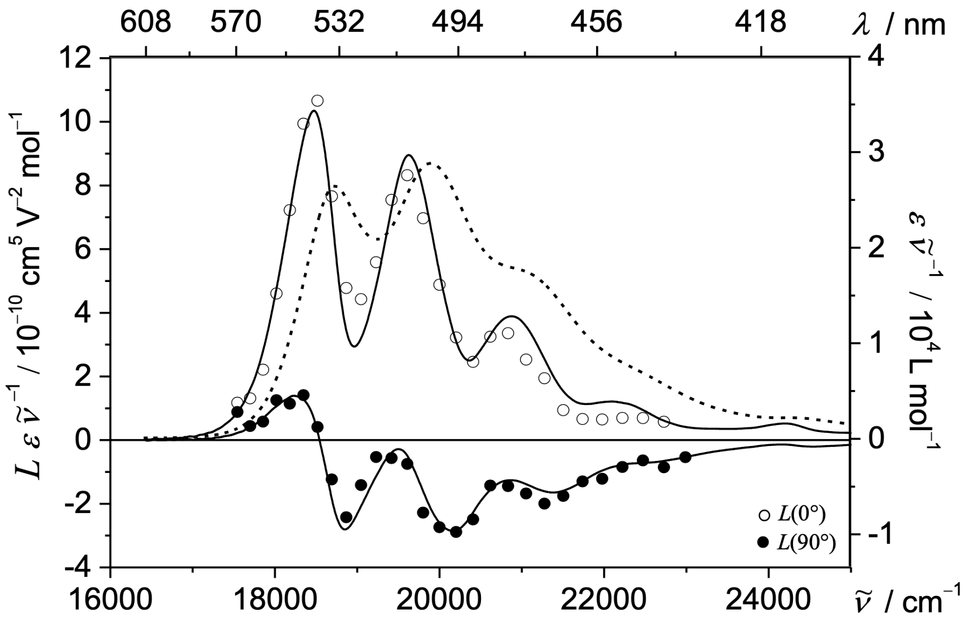

Fig. S6: UV/Vis absorption (dashed line) and EOA (0°: open symbol; 90°: solid symbol) spectra as well as the multi linear regressions (solid line) of **DCBT** measured in methylcyclohexane at 298 K.

Table S3: Fluorescence quantum yields of **DCBT** in different solvents and temperatures ($c \approx 10^{-7}$ M).

Solvent	Temperature/°C	Φ_{fl}/%
CH_2Cl_2	20	17
	−25	42
	−78	54
$CHCl_3$	20	15
	−25	46
MCH	20	1
	−25	4

Table S4: Summary of the spectroscopic data of **DCBT** in different solvents at 293 K ($c \approx 10^{-5}$ M).

Solvent	λ_{max}/nm	λ_{em}/nm	$\epsilon/10^3$ L·mol^{-1} cm^{-1}	μ_{eg}/D	Stokes shift/cm^{-1}
MCH	502 (534)^a	546	58 (50)^a	9.73	400
Tol	549	579	45	9.04	900
$CHCl_3$	561	598	56	9.73	1100
CH_2Cl_2	559	606	70	10.8	1400
MeCN	557	623	70	11.1	1900
DMSO	576	637	53	9.78	1700

^{a} In MCH contrary to the other solvents the 0-1 transition has the highest intensity. For easier comparison the values for the 0-0 transition are given in brackets.

5 Rates based on harmonic approximation and Fermi's Golden Rule

5.1 Fluorescence Rates

In the Born-Oppenheimer approximation the wave function of the initial and final states can be separated into an electronic and vibrational part

$$|i\rangle = |\Psi_i\rangle|\Theta_i\rangle \quad |f\rangle = |\Psi_f\rangle|\Theta_f\rangle, \tag{1}$$

with Ψ being the electronic wave function and Θ the vibrational one.

The potential energy surfaces of the S_0 and S_1 states are approximated to be harmonic and have the same frequencies ω_i and normal modes. The only difference is a horizontal shift of the equilibrium geometry ΔQ_i for each mode $i = 1, \dots, N$ in normal mode coordinates and a vertical shift to higher energy by the adiabatic excitation energy ΔE.

We assume that the initial state for all transitions is always the lowest vibrational level in S_1 and that contributions from higher vibrational levels and higher excited state can be neglected (This equivalent to setting the temperature to $T = 0$ K). In the initial vibrational wave function therefore all oscillators are in the ground state:

$$|\Theta_i\rangle = |\vec{0}'\rangle = |0'_1\rangle \otimes |0'_2\rangle \otimes \cdots \otimes |0'_N\rangle \tag{2}$$

The apostrophe indicates that the harmonic oscillator wavefunctions on S_1 are shifted relative to those on S_0, i.e. $\phi'(Q_i) = \phi(Q_i - \Delta Q_i)$. The final vibrational state on S_0 is

$$|\Theta_f\rangle = |\vec{m}\rangle = |m_1\rangle \otimes |m_2\rangle \otimes \cdots |m_N\rangle \tag{3}$$

where the vector $\vec{m}$ gives the number of phonons in each mode. Only those final states are allowed where the vibrational energy $E_{\vec{m}} = \sum_{i=1}^{N} m_i\omega_i$ is smaller than the adiabatic excitation energy ΔE, since otherwise no photon can be emitted.

The overlap squared between the vibrational ground state on S_1 and the final vibrational state with $\vec{m}$ phonons on S_0 is given by the product of the Franck-Condon factors for each mode,

$$|\langle 0'_i|m_i\rangle|^2 = \left| \int_{-\infty}^{+\infty} \phi_0(Q_i - \Delta Q_i)\phi_m(Q_i)dQ_i \right|^2 \tag{4}$$

$$= \frac{S_i^{m_i} e^{-S_i}}{m_i!}, \tag{5}$$

where we have defined the Huang-Rhys factor for mode i in terms of the displacement ΔQ_i as

$$S_i = \frac{1}{2}\Delta Q_i^2 \omega_i. \tag{6}$$

The overlap between the initial and final vibrational wavefunctions is given by the product of Franck-Condon factors:

$$F_{\vec{m}} = \left|\langle\vec{0}'|\vec{m}\rangle\right|^2 = \prod_{i=1}^{N} \frac{S_i^{m_i} e^{-S_i}}{m_i!} \tag{7}$$

The radiative rate increases as the 3rd power of the emission energy and 2nd power of the transition dipole moment $\vec{\mu}_{eg}$:

$$k_{\text{rad}} = u_{\text{rad}} |\vec{\mu}_{eg}|^2 \sum_{\vec{m}} (\Delta E - E_{\vec{m}})^3 F_{\vec{m}} \Theta(\Delta E - E_{\vec{m}}) \tag{8}$$

The Heaviside function $\Theta(\cdot)$ ensures that the energy difference between the initial state and final state is positive, so that a photon can be emitted. The factor $u_{\text{rad}} = 2.142 \times 10^{10}\text{s}^{-1}$ converts the rates from atomic units to s^{-1} . Summing over all vibrational states $\vec{m}$ is a difficult combinatorial problem, since the number of states grows exponentially with the number of atoms. We follow the procedure proposed in Ref. [16] and group the vibrational states into classes depending on the number of simultaneously excited modes. Limiting the sum to the first few classes $C_1, C_2, \ldots, C_{n_{\max}}$ the computational effort can be kept low while including those states that contribute most to the radiative rate. The convergence is monitored via the sum of Franck-Condon factors, $\sum_{\vec{m}} F_{\vec{m}}$, which should equal 1 if the sum is complete.

If the vibrational structure does not change at all between the S_0 and S_1 minima, only the 0-0 vibrational transition has non-vanishing Franck-Condon factors, $F_{\vec{m}} = \delta_{\vec{m},\vec{0}}$, and eqn. 8 simplifies to Einstein's expression for the spontaneous emission rate, $k_{\text{rad}}^{\text{Einstein}} = u_{\text{rad}} |\vec{\mu}_{eg}|^2 \Delta E^3$.

5.2 Internal Conversion Rates - Time-Independent

Nonadiabatic transitions are mediated by the operator $\mathcal{T}$ for the nuclear kinetic energy

$$\begin{aligned} V_{if} &= \langle\Psi_i|\langle\Theta_i|\mathcal{T}|\Psi_f\rangle|\Theta_f\rangle \\ &\approx -\sum_{j=1}^{N} \langle\Psi_i|\frac{\partial\Psi_f}{\partial Q_j}\rangle\langle\Theta_i|\frac{\partial\Theta_f}{\partial Q_j}\rangle \end{aligned} \tag{9}$$

where terms like $\langle\Psi_i|\frac{\partial^2}{\partial Q_j^2}\Psi_f\rangle$ have been neglected. The electronic derivative coupling vector is computed in Cartesian coordinates x, so we need to transform it to normal mode coordinates, $\langle\Psi_i|\frac{\partial\Psi_f}{\partial Q_j}\rangle = \sum_k \langle\Psi_i|\frac{\partial\Psi_f}{\partial x_k}\rangle\frac{\partial x_k}{\partial Q_j}$.

For non-radiative transitions we also need the nuclear part of the nonadiabatic coupling vector, i.e.

$$\langle\Theta_i|\frac{\partial\Theta_f}{\partial Q_j}\rangle = \prod_{\substack{k=1,\\k\neq j}}^{N} \langle 0'_k|m_k\rangle\langle 0'_j|\frac{\partial}{\partial Q_j}|m_j\rangle, \tag{10}$$

The physical constants that make up the prefactor can be found in eqn. 4.18 of Ref. [31]

which is derived in the appendix A. After defining the scalar product between the electronic and the nuclear nonadiabatic coupling vectors as

$$
\begin{aligned}
N_{\vec{m}} &= \sum_{j=1}^{N} \langle \Psi_i | \frac{\partial \Psi_f}{\partial Q_j} \rangle \cdot \frac{\langle 0'_j | \frac{\partial}{\partial Q_j} | m_j \rangle}{\langle 0'_j | m_j \rangle} \\
&= \sum_{j=1}^{N} \langle \Psi_i | \frac{\partial \Psi_f}{\partial Q_j} \rangle \sqrt{\omega_j} \left(\frac{m_j}{\sqrt{2S_j}} - \sqrt{\frac{S_j}{2}} \right)
\end{aligned}
\tag{11}
$$

the harmonic part of the internal conversion rate can be expressed as

$$
k_{\mathrm{ic}}^{\mathrm{harm}} = u_{\mathrm{ic}} \sum_{\vec{m}} F_{\vec{m}} N_{\vec{m}}^2 \delta \left(\Delta E - E_{\vec{m}} \right). \tag{12}
$$

The factor $u_{\mathrm{ic}} = 2.598 \times 10^{17} \mathrm{s}^{-1}$ converts the rate to s^{-1} when all other quantities are given in atomic units.

The δ-function enforces energy conservation. Only transitions to states with the same energy are allowed. These are highly excited vibrational states with vibrational energies close to the adiabatic excitation energy, $E_{\vec{m}} \approx \Delta E$. Because of the discrete nature of the vibrational states, exact alignment of initial and final vibrational states would be required to satisfy energy conservation. However, interaction with the environment leads to some broadening of the levels so that transitions are allowed within a narrow window of energies. To account for this we replace the delta function by a Gaussian with finite width Γ:

$$
\delta(\Delta E - E_{\vec{m}}) \approx \frac{1}{\sqrt{2\pi\Gamma^2}} \exp \left(-\frac{1}{2} \left(\frac{\Delta E - E_{\vec{m}}}{\Gamma} \right)^2 \right) \tag{13}
$$

The width of the energy window is taken as the average spacing between vibrational levels, $\Gamma = \frac{1}{N} \sum_i \omega_i$.

Reaching convergence proves to be much more difficult for the internal conversion rate than for the radiative rate, since the states that contribute most lie at high energies where the density of states is extremely large. There is no criterion such as the sum of Franck-Condon (FC) factors for radiative rates for assessing the convergence of IC rates. To be able to extend the summation to high classes C_n we have to cull the number of states within each class. To this end we select those normal modes have either larger nonadiabatic couplings (the so-called promoting modes) and/or large Franck-Condon factors. The normal modes are sorted in this way and the number of modes is reduced successively until the number of integrals that have to be considered falls below a manageable threshold. Since all rates are positive numbers, the rates obtained for a reduced subset of vibrational states are only a lower bound. In this way one can increase the maximum number of classes C_n and the number of integral per class until the IC rate stops changing greatly.

5.3 Internal Conversion Rates - Time-Dependent

The sum-over-states or time-independent approach of the previous section converges only very slowly. Therefore time-dependent approaches have been developed[32–34], where the infinite sums can be performed analytically at the expense of having to propagate in time. The rate for internal conversion is then obtained by a discrete Fourier transformation. For the adiabatic shift model - initial and final states only differ in the equilibrium geometry - the time-dependent formalism takes a simple form, which is presented here for completeness.

The δ-function for enforcing energy conversion in eqn. 12 can be expressed by its Fourier transform

$$\delta(\Delta E - E_{\vec{m}}) = \frac{1}{2\pi} \int_{-\infty}^{+\infty} dt \exp(\imath(\Delta E - E_{\vec{m}})t), \tag{14}$$

so that the non-radiative rate becomes

$$k_{\text{ic}}^{\text{harm}} = \frac{1}{2\pi} \int_{-\infty}^{\infty} e^{\imath \Delta E t} \left\{ u_{\text{ic}} \sum_{\vec{m}} F_{\vec{m}} N_{\vec{m}}^2 e^{-\imath E_{\vec{m}} t} \right\} dt \tag{15}$$

Now we introduce the abbreviations

$$A_j = \langle \Psi_i | \frac{\partial \Psi_f}{\partial Q_j} \rangle \sqrt{\frac{\omega_j}{2 S_j}} \tag{16}$$

$$B_j = -\langle \Psi_i | \frac{\partial \Psi_f}{\partial Q_j} \rangle \sqrt{\frac{\omega_j S_j}{2}} \tag{17}$$

so that eqn. 11 can be written as

$$N_{\vec{m}} = \sum_j (A_j m_j + B_j) \tag{18}$$

The Fourier transform of the non-radiative rate becomes then

$$\begin{aligned} \tilde{k}_{\text{ic}}(t) &= u_{\text{ic}} \sum_{\vec{m}} F_{\vec{m}} N_{\vec{m}}^2 e^{-\imath E_{\vec{m}} t} \\ &= u_{\text{ic}} \sum_{\vec{m}} \prod_{k=1}^{N} \frac{1}{m_k!} S_k^{m_k} e^{-S_k - \imath \omega_k t m_k} \sum_{i=1}^{N} (A_i m_i + B_i) \sum_{j=1}^{N} (A_j m_j + B_j) \end{aligned} \tag{19}$$

We introduce the abbreviation

$$G_m^{(i)} = e^{-S_i} \frac{1}{m!} \left(S_i e^{-\imath \omega_i t} \right)^m, \tag{20}$$

write out the sum over vibrational quantum numbers

$$\sum_{\vec{m}} = \sum_{m_1=0}^{\infty} \sum_{m_2=0}^{\infty} \dots \sum_{m_i=0}^{\infty} \dots \sum_{m_N=0}^{\infty} \tag{21}$$

and group the factors $G_{m_i}^{(i)}$ with their respective sums. This gives

$$\begin{aligned} \tilde{k}_{\mathrm{nr}}(t) = u_{\mathrm{ic}} & \sum_{i=1}^{N} \sum_{j \neq i}^{N} \sum_{m_1=0}^{\infty} G_{m_1}^{(1)} \dots \\ & \sum_{m_{i-1}=0}^{\infty} G_{m_{i-1}}^{(i-1)} \sum_{m_i=0}^{\infty} G_{m_i}^{(i)} (A_i m_i + B_i) \sum_{m_{i+1}=0}^{\infty} G_{m_{i+1}}^{(i+1)} \dots \\ & \sum_{m_{j-1}=0}^{\infty} G_{m_{j-1}}^{(j-1)} \sum_{m_j=0}^{\infty} G_{m_j}^{(j)} (A_j m_j + B_j) \sum_{m_{j+1}=0}^{\infty} G_{m_{j+1}}^{(j+1)} \dots \\ & \sum_{m_N=0}^{\infty} G_{m_N}^{(N)} \\ + u_{\mathrm{ic}} & \sum_{i=1}^{N} \sum_{m_1=0}^{\infty} G_{m_1}^{(1)} \dots \\ & \sum_{m_{i-1}=0}^{\infty} G_{m_{i-1}}^{(i-1)} \sum_{m_i=0}^{\infty} G_{m_i}^{(i)} (A_i m_i + B_i)^2 \sum_{m_{i+1}=0}^{\infty} G_{m_{i+1}}^{(i+1)} \dots \\ & \sum_{m_N=0}^{\infty} G_{m_N}^{(N)}. \end{aligned} \tag{22}$$

The double sums over i and j were split into sums where $i \neq j$ and $i = j$. We introduce the abbreviation

$$X_i(t) = S_i e^{-\imath \omega_i t} \tag{23}$$

The sums over m are performed by recognizing the Taylor expansion of the exponential function, $\sum_{m=0} x^m/m! = e^x$, $\sum_{m=0} m/m! x^m = x e^x$ and $\sum_{m=0} m^2/m! x^m = (x^2 + x) e^x$ for $x = S e^{-\imath \omega t}$:

$$\sum_{m=0}^{\infty} G_m^{(i)} = e^{-S_i} \sum_{m=0}^{\infty} \frac{1}{m!} X_i^m = e^{-S_i + X_i} \tag{24}$$

and similarly

$$
\begin{aligned}
&\sum_{m=0}^{\infty} G_m^{(i)} (A_i m + B_i) \\
&\qquad = e^{-S_i} \left\{ A_i \sum_{m=0}^{\infty} \frac{m}{m!} X_i^m + B_i \sum_{m=0}^{\infty} \frac{1}{m!} X_i^m \right\} \\
&\qquad = e^{-S_i + X_i} (A_i X_i + B_i)
\end{aligned}
\tag{25}
$$

and

$$
\begin{aligned}
&\sum_{m=0}^{\infty} G_m^{(i)} (A_i m + B_i)^2 \\
&\qquad = e^{-S_i} \sum_{m=0}^{\infty} \frac{X_i^m}{m!} \left\{ A_i^2 m^2 + 2 A_i B_i m + B_i^2 \right\} \\
&\qquad = e^{-S_i + X_i} \left\{ A_i^2 X_i^2 + (2 A_i B_i + A_i^2) X_i + B_i^2 \right\}
\end{aligned}
\tag{26}
$$

Substituting eqns. 24, 25 and 26 into 22 gives finally

$$
\begin{aligned}
\tilde{k}_{\mathrm{ic}}(t) = u_{\mathrm{ic}} & \left(\prod_{k=1}^{N} e^{-S_k + X_k(t)} \right) \\
\times & \left\{ \sum_{i=1}^{N} \sum_{j=1}^{N} (A_i X_i(t) + B_i)(A_j X_j(t) + B_j) + \sum_{i=1}^{N} A_i^2 X_i(t) \right\}
\end{aligned}
\tag{27}
$$

Since vibrational energy levels are discrete, energy conservation would require perfect energetic alignment of the initial and final vibrational states. Initial and final states will only accidentally have exactly the same energy when the vibrational frequencies are commensurable. Therefore we need to relax energy conservation a little bit by replacing the δ-function with the Gaussian broadening function of eqn. 13. The physical justification is that the coupling to an environment broadens the vibrational levels and allows transitions between levels which are close so that their line shape functions overlap.

Using the Fourier transform of a Gaussian the broadened line shape function can be expressed as

$$
\delta(\Delta E - E_{\vec{m}}) \approx \frac{1}{2\pi} \int_{-\infty}^{\infty} dt \exp\left(-\frac{1}{2} \Gamma^2 t^2 \right) \exp(\imath (\Delta E - E_{\vec{m}}) t). \tag{28}
$$

If we use this definition instead of the δ-function in eqn. 15 the non-radiative rate as a function of the excitation energy becomes

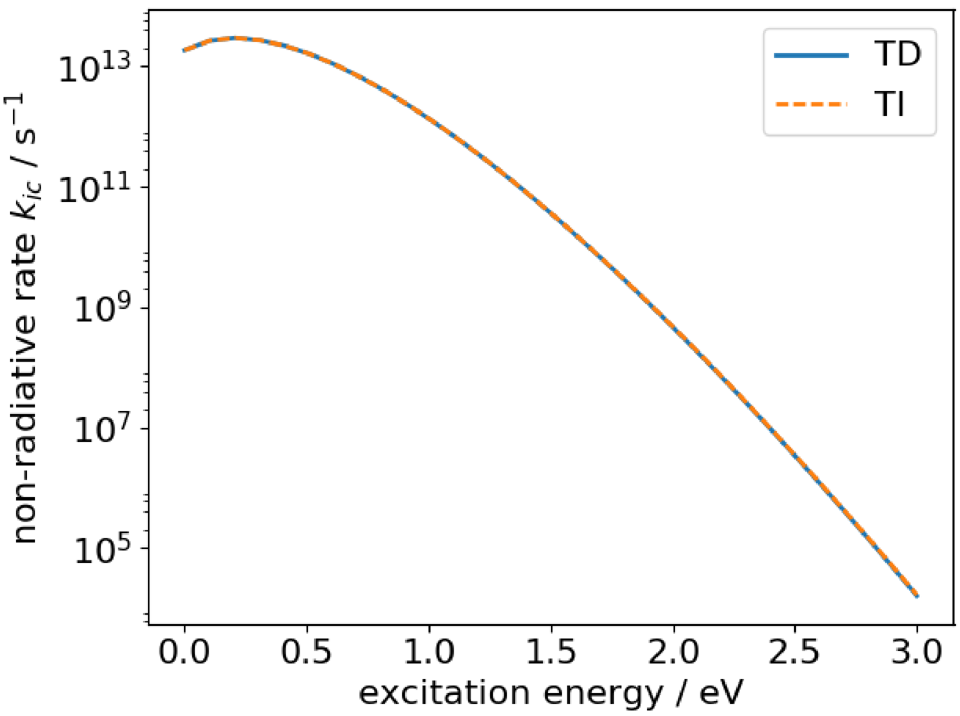

Fig. S7: Comparison of non-radiative rates k_{ic} as functions of excitation energy computed with the time-dependent (TD) and time-independent (TI) formalism for a fictitious system with 3 vibrational modes. The frequencies, Huang-Rhys factors and non-adiabatic couplings of the model were set to $\omega_1 = 1000$ cm^{-1}, $\omega_2 = 1111$ cm^{-1}, $\omega_2 = 2100$ cm^{-1}; $S_1 = 0.1$, $S_2 = 0.01$, $S_3 = 0.5$; $\langle \Psi_i | \partial \Psi_f / \partial Q_1 \rangle = -0.01$, $\langle \Psi_i | \partial \Psi_f / \partial Q_2 \rangle = 0.03$, $\langle \Psi_i | \partial \Psi_f / \partial Q_3 \rangle = 0.005$. The parameters for the discrete Fourier transform were, $N_t = 2^{16}$ sample points and a maximum propagation time of $T = 4000$ fs. At an excitation energy of 1 eV the rate amounts to $k_{\text{ic}}^{\text{harm}} = 1.36 \times 10^{12}$ s^{-1}. The small discrepancy between TD and TI formalism at high energies is due to the finite energy resolution of the discrete Fourier transform.

$$k_{\text{ic}}^{\text{harm}}(\Delta E) = \frac{1}{2\pi} \int_{-\infty}^{\infty} e^{\imath \Delta E t} \left\{ e^{-\frac{1}{2}\Gamma^2 t^2} \tilde{k}_{ic}(t) \right\} dt. \tag{29}$$

$\tilde{k}_{\text{ic}}(t)$ is evaluated on an equidistant grid covering the interval $[-T, T]$ and the non-radiative rate at the given excitation energy ΔE is then obtained by interpolating the discrete Fourier transform in eqn. 29. The time-dependent formalism is much more efficient than the time-independent one at the cost that the information about which vibrational levels contribute most to the rate is lost. Except for the discretization error, both formalisms, if converged, give the same results (see Fig. S7).

5.3.1 Energy gap law - saddle point integration To derive an energy gap law from eqn. 15 some simplifying assumptions need to be made: (1) The frequencies and Huang-Rhys factors of all modes are replaced by their average values, $\omega_{\text{eff}} = \sum_{i=1}^{N} \omega_i \frac{C_i}{\sum_{j=1}^{N} C_j}$ is the effective vibrational mode and $S = N^{-1} \sum_{i=1}^{N} S_i$. (2) The $P < N$ modes which have non-vanishing non-adiabatic couplings are called promoting modes. The coupling per mode is replaced

by the average value, so that the total length of the non-adiabatic coupling vector remains unchanged: $\left|\langle\Psi_i|\frac{\partial\Psi_f}{\partial Q}\rangle\right|^2_{\text{avg}} = P^{-1}\sum_{i=1}^{P}\left|\langle\Psi_i|\frac{\partial\Psi_f}{\partial Q_i}\rangle\right|^2$. Substituting eqn. 27 into eqn. 15 and using the averaged quantities, the non-radiative rate becomes

$$\begin{aligned} k_{\text{ic}}^{\text{harm}} = & \frac{u_{ic}}{4\pi}\left|\langle\Psi_i|\frac{\partial\Psi_f}{\partial\vec{Q}}\rangle\right|^2 \omega_{\text{eff}} \\ & \times\Big\{PS\int_{-\infty}^{\infty}\exp\left(\imath\Delta Et + NS(e^{-\imath\omega_{\text{eff}}t}-1)\right)dt \\ & -(2PS-1)\int_{-\infty}^{\infty}\exp\left(\imath[\Delta E-\omega_{\text{eff}}]t + NS(e^{-\imath\omega_{\text{eff}}t}-1)\right)dt \\ & +PS\int_{-\infty}^{\infty}\exp\left(\imath[\Delta E-2\omega_{\text{eff}}]t + NS(e^{-\imath\omega_{\text{eff}}t}-1)\right)dt\Big\} \end{aligned} \tag{30}$$

The three integrals all have the form

$$\int_{-\infty}^{\infty}\exp(f(t))dt \tag{31}$$

with

$$f(t) = \imath\varepsilon t + NS(e^{-\imath\omega_{\text{eff}}t}-1) \tag{32}$$

for $\varepsilon = \Delta E, \Delta E - \omega_{\text{eff}}, \Delta E - 2\omega_{\text{eff}}$. Integrals of this type can be approximated by saddle point integration (see chapter 10.5 in Ref.[35]). The largest contribution to the integral comes from the region around the stationary points of $f(t)$ (where $f'(t_0) = 0$). After expanding the exponent to quadratic order around the stationary point, the resulting Gaussian integral can be solved:

$$\begin{aligned} \int_{-\infty}^{\infty} e^{f(t)}dt &\approx \int_{-\infty}^{\infty}\exp\left(f(t_0) + \underbrace{f'(t_0)}_{=0}(t-t_0) + \frac{1}{2}f''(t_0)(t-t_0)^2\right)dt \\ &\overset{x=t-t_0}{=} e^{f(t_0)}\int_{-\infty}^{\infty}\exp\left(-\frac{1}{2}[-f''(t_0)]x^2\right)dx \\ &= e^{f(t_0)}\sqrt{\frac{2\pi}{(-f''(t_0))}} \end{aligned} \tag{33}$$

The derivatives of $f(t)$ are

$$f'(t) = i\varepsilon - \imath\omega_{\text{eff}}NSe^{-\imath\omega_{\text{eff}}t} \tag{34}$$

$$f''(t) = -\omega_{\text{eff}}^2 NSe^{-\imath\omega_{\text{eff}}t} \tag{35}$$

$f'(t)$ has a single root at

$$\imath t_0 = \frac{1}{\omega_{\text{eff}}}\log\left(\frac{\omega_{\text{eff}}NS}{\varepsilon}\right) \tag{36}$$

which corresponds to a maximum since

$$f''(t_0) = -\omega_{\text{eff}}\varepsilon \quad < 0. \tag{37}$$

The value of the exponent at the maximum $t = t_0$ is

$$f(t_0) = -\frac{\varepsilon}{\omega_{\text{eff}}}\left(\log\left(\frac{\varepsilon}{\omega_{\text{eff}} NS}\right) - 1\right) - NS. \tag{38}$$

Putting everything together the saddle point approximation for our integral reads:

$$\int_{-\infty}^{\infty} e^{f(t)} dt \approx \exp\left(-\frac{\varepsilon}{\omega_{\text{eff}}}\left(\log\left(\frac{\varepsilon}{\omega_{\text{eff}} NS}\right) - 1\right) - NS\right)\sqrt{\frac{2\pi}{\omega_{\text{eff}}\varepsilon}} \tag{39}$$

Using this approximation to evaluate the three integrals in eqn. 30 we obtain the non-radiative rate in the saddle point approximation:

$$\begin{aligned} k_{\text{ic}}^{\text{harm}} = & \frac{u_{ic}}{2\sqrt{2\pi}} \left| \langle \Psi_i | \frac{\partial \Psi_f}{\partial \vec{Q}} \rangle \right|^2 e^{-NS} \\ & \times PS \left\{ I\left(\frac{\Delta E}{\omega_{\text{eff}}}\right) - (2 - \frac{1}{PS}) I\left(\frac{\Delta E}{\omega_{\text{eff}}} - 1\right) + I\left(\frac{\Delta E}{\omega_{\text{eff}}} - 2\right) \right\} \end{aligned} \tag{40}$$

where the function $I(x)$ of the the dimensionless argument x is defined as:

$$I(x) = \sqrt{\frac{1}{x}} \exp\left(-x\left[\log\left(\frac{x}{NS}\right) - 1\right]\right) \tag{41}$$

According to eqn. 40, the non-radiative rate is proportional to the electronic non-adiabatic coupling matrix element, decreases exponentially with the displacement between ground and excited state minima (because of the exponential factor e^{-NS} and the relation $S = \frac{1}{2}\Delta Q^2 \omega_{\text{eff}}$), which is surprising, and has a seemingly complex dependence on ΔE because of the interference between the three I-functions at slightly shifted arguments. For adiabatic excitation energies that are large relative to the average vibrational frequency, $\Delta E \gg \omega_{\text{eff}}$, one recognizes in eqn. 40 a term that is similar to a finite difference approximation for the second derivative of I,

$$I(x) - 2I(x-1) + I(x-2) \approx I''(x-1) \qquad \text{for } \; x \gg 1, \tag{42}$$

so that eqn. 40 simplifies to

$$\begin{aligned} k_{\text{ic}}^{\text{harm}} \approx & \frac{u_{ic}}{2\sqrt{2\pi}} \left| \langle \Psi_i | \frac{\partial \Psi_f}{\partial \vec{Q}} \rangle \right|^2 \\ & \times e^{-NS} \times \left\{ I\left(\frac{\Delta E}{\omega_{\text{eff}}} - 1\right) + PS \; I''\left(\frac{\Delta E}{\omega_{\text{eff}}} - 1\right) \right\} \end{aligned} \tag{43}$$

This is the energy gap law (cf. Ref.[36]) at $T = 0$ K. For large adiabatic excitation energies the non-radiative rate decreases faster than exponentially, because of the behaviour of $I(x)$ for $x \to \infty$.

Fig. S8 shows the energy dependence of the non-radiative rate according to the energy gap for the merocyanine dye.

Eqn. 43 is similar to the energy gap law derived by Englman and Jortner (eqn. 5.4 in Ref.[36]) in the weak coupling limit for $T = 0$ K, which in our notation would read

$$k_{\text{ic}}^{\text{harm}} \approx \frac{u_{\text{ic}}}{2\sqrt{2\pi}} \left| \langle \Psi_i | \frac{\partial \Psi_f}{\partial \vec{Q}} \rangle \right|^2 e^{-NS} I\left(\frac{\Delta E}{\omega_{\text{eff}}}\right) \tag{44}$$

Our result differs from theirs primarily by the second derivative of $I(x)$. This additional term arises because we take into account the scalar product between the electronic and nuclear non-adiabatic coupling vectors in eqn. 11, while Englman and Jortner make the approximation $N_{\vec{m}}^2 \propto C^2$, lumping the contributions of different vibrational states to the non-adiabatic coupling into a single constant C. This simplifies their problem so that they can use tricks originally developed to calculate the shape of absorption curves[37,38] to derive the non-radiative rate at non-zero temperature (eqn. (5.12) in Ref.[36]). The more complicated nature of eqn. 11 precludes us from using the same techniques. However, a back-of-the-envelope calculation shows that temperature effects on the non-radiative rate in the harmonic approximation are likely negligible compared to the errors introduced by the harmonic approximation itself. At room temperature the thermal energy amounts to $\beta^{-1} = k_B \times 300\ K = 209\ \text{cm}^{-1}$ compared to the energy of a C=C stretch vibration (a typical promoting mode) in the range $\hbar\omega_{\text{eff}} = 1400 - 1600\ \text{cm}^{-1}$, which gives $\beta\hbar\omega_{\text{eff}} \approx 7$. The probability that the promoting mode in the initial state is excited at room temperature is negligible:

$$\begin{aligned} p(n \geq 1) &= \sum_{n=1}^{\infty} p(n) = \frac{\sum_{n=1}^{\infty} e^{-\beta\hbar\omega_{\text{eff}}(n+\frac{1}{2})}}{\sum_{n=0}^{\infty} e^{-\beta\hbar\omega_{\text{eff}}(n+\frac{1}{2})}} = e^{-\beta\hbar\omega_{\text{eff}}} \\ &\approx 0.0009, \end{aligned} \tag{45}$$

Vibrational modes with lower frequencies can be excited thermally, but their contribution to the non-adiabatic coupling vector is smaller than that of the promoting modes.

5.4 Notes on the saddle point approximation

To understand why and in which cases the saddle point approximation works, one has to remember that integrals of analytic functions in the complex plane depend only on the endpoints. With reference to Fig. S9 this means

$$\int_A^B g(z)dz = \int_{C_1} g(z)dz = \int_{C_2} g(z)dz. \tag{46}$$

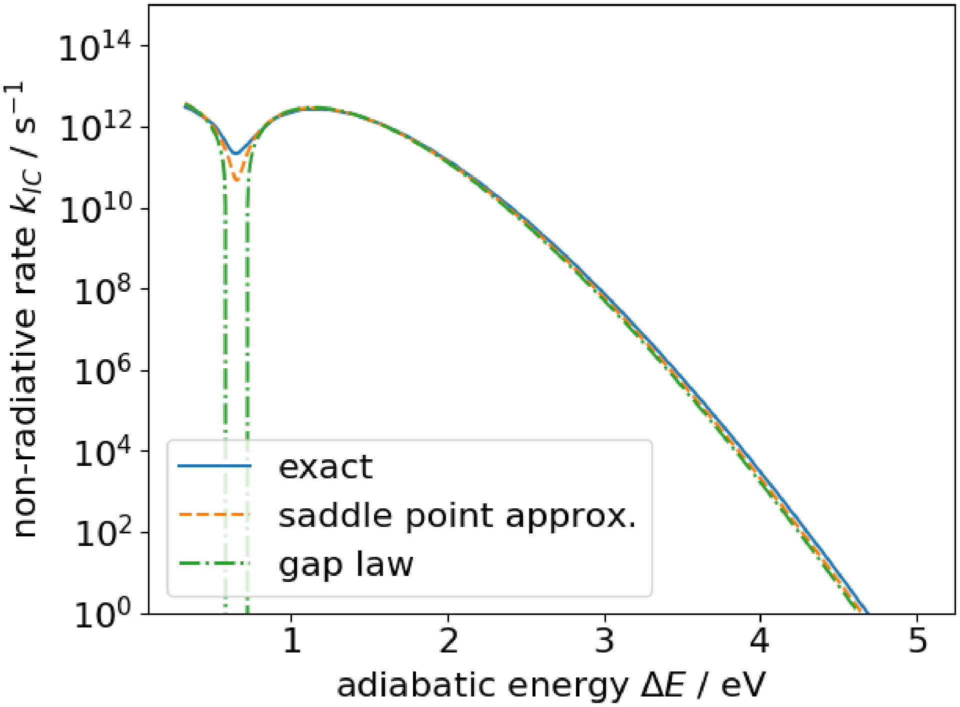

Fig. S8: **Energy gap law** for non-radiative rates. Average frequencies and Huang-Rhys factors from the merocyanine dye were used. Non-radiative rates are shown for different approximations: numerically exact integrals by Fourier transformation according to eqn. 30 (solid blue, convolved with a Gaussian of exponent $\Gamma = \frac{1}{2}\omega_{\text{eff}}$), saddle point approximation according to eqn. 40 (dashed orange) and the energy gap law according to eqn. 43 (dotted green). Interestingly there is a dip at ≈ 0.8 eV where k_{ic} has a local minimum.

We need to solve the following integral of an analytic function over the real line

$$I = \int_{-\infty}^{\infty} e^{-\frac{1}{2}\gamma t^2} e^{f(t)} \qquad \text{with} \qquad f(t) = \imath\varepsilon t + NS(e^{-\imath\omega_{\text{eff}}t} - 1) \tag{47}$$

where $\gamma > 0$ is a Gaussian broadening parameter. $f(t)$ can be split into real and imaginary parts:

$$e^{f(t)} = e^{x(t)} e^{\imath y(t)} \tag{48}$$

The largest contributions to the integral come from regions where (1) $x(t)$ has a maximum, i.e. $x'(t_0) = 0$ and (2) where the imaginary part is stationary, i.e. $y'(t_0) = 0$, so that the phase does not change appreciably, since fast oscillations would cancel. Conditions (1) and (2) cannot be fulfilled simultaneously unless t_0 is allowed to be complex. Then the separate conditions for real and imaginary parts can be combined into a single equation

$$f'(z_0) = 0 \tag{49}$$

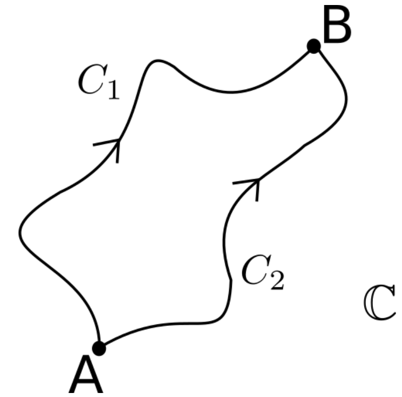

Fig. S9: Integration paths in the complex plane.

which leads to

$$z_0 = -\frac{\imath}{\omega_0} \log\left(\frac{\omega_{\text{eff}} NS}{\varepsilon}\right) \tag{50}$$

Since z_0 is imaginary and does not lie on the real axis, the point is not traversed in the line integral of eqn. 47. However, if the path is deformed so that it passes through z_0 (see Fig.S10), the value of the integral is not changed, but the integrand oscillates less and is more strongly localized, so that it can be approximated by a Gaussian function (see Fig. S11). The parametrization of the new path is

$$z(t) = z_0 + t \qquad t \in [-\infty, +\infty] \tag{51}$$

Since the endpoints are $-\infty$ and $+\infty$, it is fine to neglect the curvature. The quadratic expansion of the exponent around z_0 is

$$f(z) \approx f(z_0) + \underbrace{f'(z_0)}_{=0}(z - z_0) - \frac{1}{2}[-f''(z_0)](z - z_0)^2 \tag{52}$$

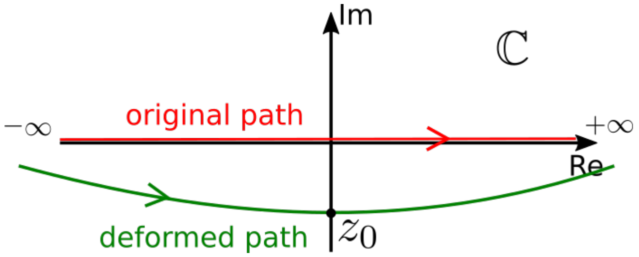

Fig. S10: Deformed path passes through z_0

The function $f(z_0 + t)$ is periodic, while the quadratic approximation has a single maximum. The broadening parameter γ is needed to ensure that only the first peak contributes to the integral (see Fig. S12). After performing the quadratic expansion we can set $\gamma = 0$, but it is important to keep in mind, that without this damping factor, the integral $I(\varepsilon)$ would

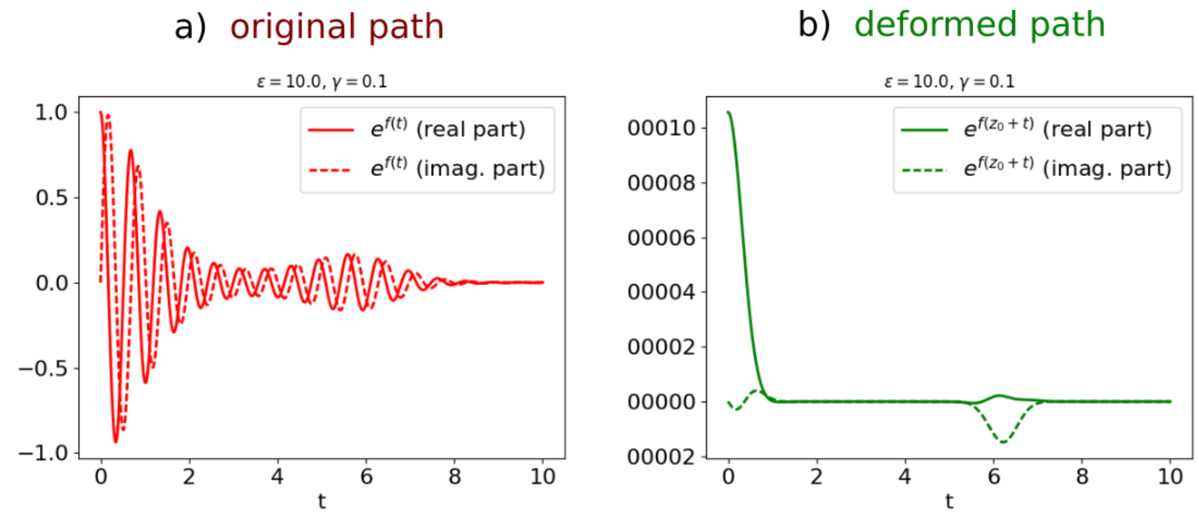

Fig. S11: (a) Along the real line $e^{f(t)}$ is a highly oscillatory function. (b) If the path is deformed so that is passes through z_0, $e^{f(t)}$ is localized around $t = 0$ and can be approximated by a Gaussian.

be a sequence of δ-peaks only envelope of which agrees with the saddle point approximation.

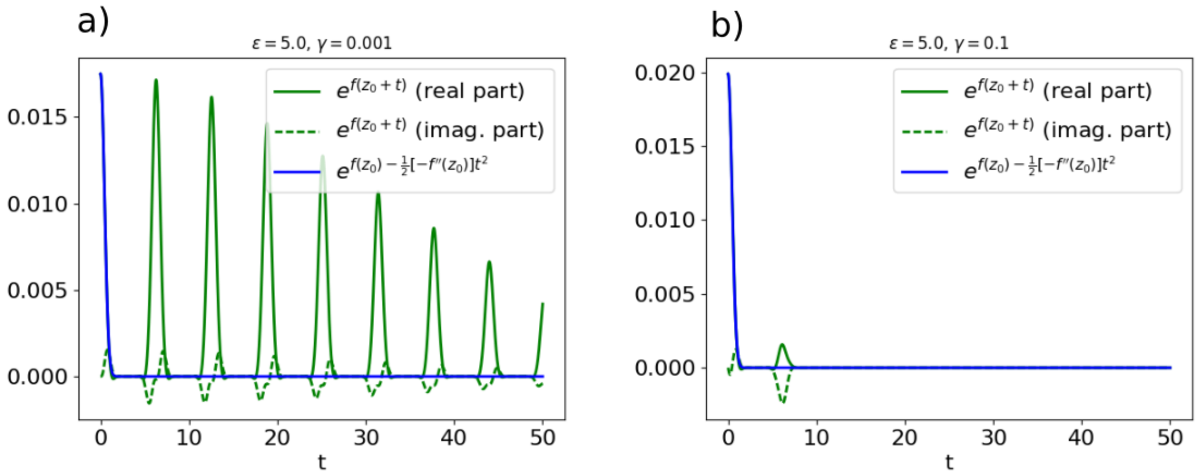

Fig. S12: $e^{f(t)}$ for (a) weak damping and (b) strong damping. If γ is chosen large enough only a single peak survives.

We conclude that the saddle point approximation

$$[h] \int_{-\infty}^{\infty} e^{f(t)} dt = \int_{-\infty}^{\infty} e^{f(z_0+t)} dt \approx e^{f(z_0)} \int_{-\infty}^{\infty} e^{-\frac{1}{2}[-f''(z_0)]t^2} dt \tag{53}$$

is asymptotically valid in the limits $\varepsilon \to \infty$ and $\gamma \to \infty$.

5.5 Nonadiabatic Couplings

Here we derive the expression for the non-adiabatic coupling between displaced harmonic oscillator eigenfunctions in terms of the Huang-Rhys factors. The indices labelling the vi-

brational modes are suppressed for clarity. The action of the momentum operator $\mathcal{P}$ on an eigenstate of the harmonic oscillator is given by[39]

$$-\imath \frac{d\phi_m(Q)}{dQ} = \mathcal{P}\phi_m \tag{54}$$

$$= \imath \sqrt{\frac{\omega_{\text{eff}}}{2}} \left(\sqrt{m+1}\phi_{m+1}(Q) - \sqrt{m}\phi_{m-1}(Q) \right) \tag{55}$$

Therefore the derivative coupling can be expressed in terms of overlap integrals to states with $m-1$ and $m+1$ phonons:

$$\begin{aligned} \langle 0' | \frac{\partial}{\partial Q} | m \rangle &= \int_{-\infty}^{\infty} \phi_0(Q - \Delta Q) \frac{d\phi_m}{dQ}(Q) dQ \\ = -\sqrt{\omega_{\text{eff}}} &\left(\sqrt{\frac{m+1}{2}} \int_{-\infty}^{\infty} \phi_0(Q - \Delta Q)\phi_{m+1}(Q) dQ \right. \\ &\left. - \sqrt{\frac{m}{2}} \int_{-\infty}^{\infty} \phi_0(Q - \Delta Q)\phi_{m-1}(Q) dQ \right) \end{aligned} \tag{56}$$

Using the definition of the Franck-Condon factors in eqn. 5 we obtain the coupling expressed via the Huang-Rhys factors:

$$\begin{aligned} \langle 0' | \frac{\partial}{\partial Q} | m \rangle &= \sqrt{\omega_{\text{eff}}} \left(\sqrt{\frac{m}{2}} \sqrt{\frac{S^{m-1}e^{-S}}{(m-1)!}} - \sqrt{\frac{m+1}{2}} \sqrt{\frac{S^{m+1}e^{-S}}{(m+1)!}} \right) \\ &= \langle 0' | m \rangle \sqrt{\omega_{\text{eff}}} \left(\frac{m}{\sqrt{2S}} - \sqrt{\frac{S}{2}} \right) \end{aligned} \tag{57}$$

6 Rates based on Kramers's Theory

For the evaluation of the Kramers's equation (see eqn. 8 in the main article) the electronic energy gap between the S_1 minimum and transition state, which connects the FC minimum and the conical intersection, is used to calculate the nonradiative rate $k_{\mathrm{ic}}^{\mathrm{CI}}$. However, our calculated activation barriers E_{A} are too high and if plugged unadulterated into Kramers's equation would lead to negligibly small internal conversion rates. Furthermore, also the prefactor might be prone to errors and therefore this fitting to the experimental values corrects both for the systematic errors in the barrier heights as well for the errors in the frequency prefactor. In view of the clear correlation between activation barriers and quantum yields, we introduce a parameter α for shifting the activation energies to a reasonable magnitude. α is fitted to minimize the difference between experimental and simulated quantum yields. The optimal value of α is obtained by finding the maximum of the sample Pearson correlation coefficient[40],

$$r_{xy} = \frac{n\sum_{i=1}^{n} x_i y_i - \sum_{i=1}^{n} x_i \sum_{i=1}^{n} y_i}{\sqrt{n\sum_{i=1}^{n} x_i^2 - \left(\sum_{i=1}^{n} x_i^2\right)^2}\sqrt{n\sum_{i=1}^{n} y_i^2 - \left(\sum_{i=1}^{n} y_i^2\right)^2}}, \tag{58}$$

between the two data sets. Here x_i, y_i are the experimental and simulated quantum yields in the different solvents and n is the number of solvents. This gives $\alpha = 0.192$ eV.

6.1 Calculation of Friction Coefficients

The microscopic friction coefficients, γ, were calculated using Stokes law:

$$\gamma = \frac{6 \cdot \pi \cdot \eta \cdot r}{m} \tag{59}$$

Here η is the dynamic viscosity of the solvent, r the radius of a single solvent molecule and m the molar mass. The radii were calculated from the macroscopic density and the molar mass by assuming that the geometry of the solvent molecule corresponds to a hard sphere. The used data in combination with the reference are given in Tab. S5.

Table S5: Dynamic viscosities (η), molecular weights (M) and densities of the used solvents. The corresponding reference is given after each value.

	η/mPa · s	M/g·mol^{-1}	ρ/g·cm^3
MCH	0.734[41]	98.19[42]	0.77[42]
Tol	0.590[43]	92.14[44]	0.86[44]
CHCl3	0.563[45]	119.37[45]	1.48[45]
CH2Cl2	0.437[46]	84.93[46]	1.33[46]
MeCN	0.350[47]	41.05[47]	0.79[47]
DMSO	2.470[48]	78.13[48]	1.10[48]

7 Onsager Solvation Model

Having established by the CDFT-CI calculations and the BLA criterion that the weight c^2 changes along the path FC $\rightarrow$ TS $\rightarrow$ MECI, we can interpret c^2 as a reaction coordinate. CDFT-CI also provides qualitative matrix elements of the x-component (along the principal axis of the molecule) of the dipole operator for the diabatic wavefunctions (in Debye):

$$\mu_{\text{neutral}} = \langle \mathbf{DA}|ex|\mathbf{DA}\rangle = 6.1 \text{ D} \tag{60}$$
$$\mu_{\text{zwitter}} = \langle \mathbf{D}^+\mathbf{A}^-|ex|\mathbf{D}^+\mathbf{A}^-\rangle = 40.5 \text{ D} \tag{61}$$
$$\mu_{\text{cross}} = \langle \mathbf{DA}|ex|\mathbf{D}^+\mathbf{A}^-\rangle = -3.7 \text{ D} \tag{62}$$

With the help of the diabatic to adiabatic transformation

$$\Psi_{\text{g}} = \sqrt{1-c^2}|\mathbf{DA}\rangle + c|\mathbf{D}^+\mathbf{A}^-\rangle \tag{63}$$
$$\Psi_{\text{e}} = c|\mathbf{DA}\rangle - \sqrt{1-c^2}|\mathbf{D}^+\mathbf{A}^-\rangle, \tag{64}$$

the permanent dipole moments of the adiabatic ground and excited state may be expressed as functions of the parameter c:

$$\mu_{\text{g}}(c) = \sqrt{1-c^2}\mu_{\text{neutral}} + c^2\mu_{\text{zwitter}} + 2c\sqrt{1-c^2}\mu_{\text{cross}} \tag{65}$$
$$\mu_{\text{e}}(c) = c^2\mu_{\text{neutral}} + (1-c^2)\mu_{\text{zwitter}} - 2c\sqrt{1-c^2}\vec{\mu}_{\text{cross}} \tag{66}$$

These qualitative dipole moments are plotted in the upper part of Fig. S13. The similarity of the curves with the quantitative dipole moments from CASSCF calculations in Fig. 4 justifies the choice of c as a reaction coordinate, but it is also obvious that the amplitude of these dipole moments is overestimated.

Now we are in a position to build a simple model that explains why the polarity of the solvent affects different points along the reaction coordinate differently. In the Onsager model[49,50], the solute is approximated by a dipole in a spherical cavity of radius a surrounded by a homogeneous dielectric medium of the relative dielectric constant ϵ_{r} (to vacuum). The dipole moment induces a polarization in the dielectric which gives rise to a reaction field $\vec{R}$, the strength of which depends on ϵ_r:

$$\vec{R} = \frac{2(\epsilon_{\text{r}}-1)}{2\epsilon_{\text{r}}+1}\frac{\mu}{a^3} \tag{67}$$

If the excited state is long-lived, the reaction field has time to adjust to the excited state dipole moment. The state-specific solvation energy is given by the interaction of the dipole moment of the ground or excited state with the respective reaction field:

$$U_{\rm sol}(c) = -\frac{1}{2}\mu \cdot \vec{R} = -\frac{\epsilon_{\rm r} - 1}{2\epsilon_{\rm r} + 1}\frac{\mu^2(c)}{a^3} \tag{68}$$

The Onsager model is only able to predict qualitative trends. For elongated planar molecules the assumption of a spherical cavity is questionable and it is not clear which value should be chosen for the cavity radius – we use a value of $a \approx 10$ Å.
The solvation energies along the reaction path according to eqn. 68 are shown on the right in Fig. S13b). For $c \approx 0$ the excited state is largely zwitterionic and strongly stabilized by polar solvents, whereas the stabilization is only small for $c \approx 1$. This leads to an increase in the activation energy and consequently a reduction in the rate for non-radiative decay through the CI.

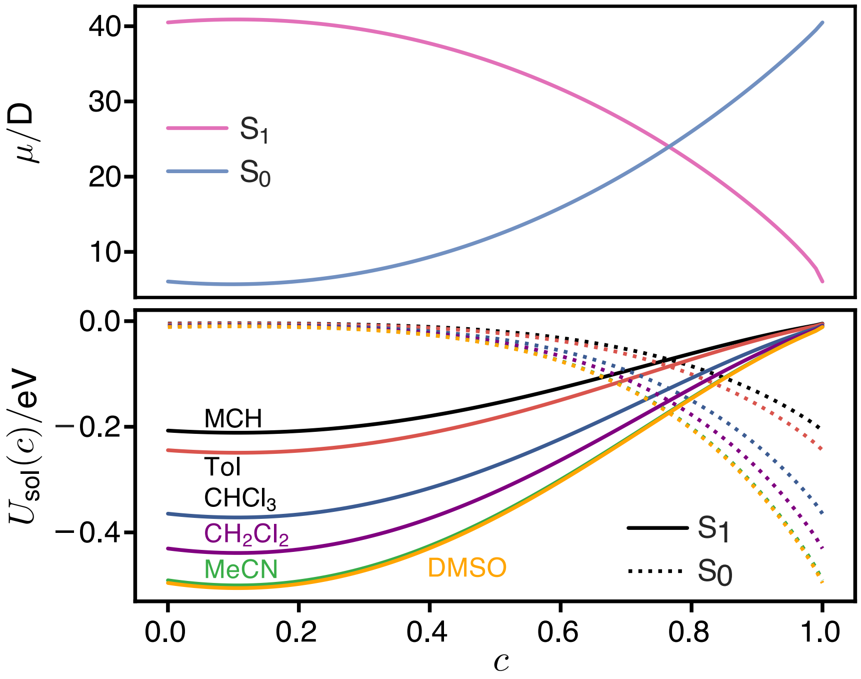

Fig. S13: (top) Permanent dipole moments in the ground and excited state along the reaction coordinate c. (bottom) State-specific solvation energy from Onsager's model for solvents of increasing polarity. The Franck-Condon point ($c \approx 0.52$) is stabilized more by a polar solvent than the transition state and the MECI ($c \approx 0.71$).

8 Transient Absorption Spectroscopy

Table S6: Overview of lifetimes resulting from global analysis of broadband ultrafast transient absorption data in various solvents. Lifetimes marked with a star symbol are larger than the maximum measured time delay and should therefore be regarded as infinite in the context of the performed measurements. Root-mean square errors obtained for global fits: $\mathrm{RMS}_{\mathrm{MCH}} = 3.65 \cdot 10^{-5}$ mOD, $\mathrm{RMS}_{\mathrm{CHCl_3}} = 4.7 \cdot 10^{-4}$ mOD and $\mathrm{RMS}_{\mathrm{MeCN}} = 2.43 \cdot 10^{-4}$ mOD.

Solvent	λ_{ex}/nm	τ_1/fs	τ_2/ps	τ_3/ps	τ_4/ps	τ_5/ns
MCH	506	135		5.3	36	10.8*
$CHCl_3$	518	586.7	3.6	169.9	834	12.5*
MeCN	514	168.6	1.3	8.5	1448.5*	

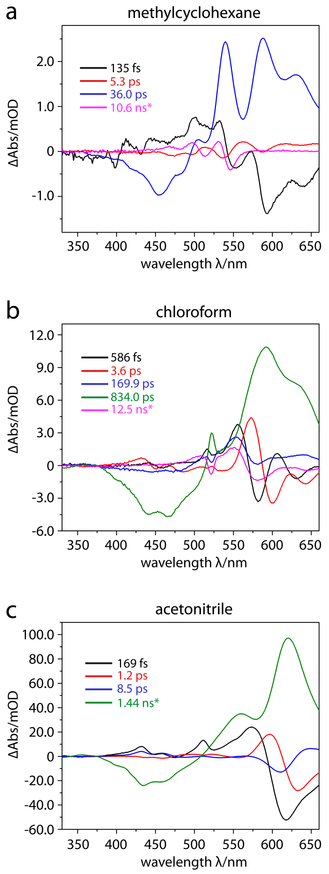

Fig. S14: Decay-associated spectra and relaxation times (included in the plots) resulting from global analysis of the transient absorption data of Fig. 7 in the main text for the three solvents: (a) Methylcyclohexane at $\lambda_{ex} = 506$ nm, (b) chloroform at $\lambda_{ex} = 518$ nm, and (c) acetonitrile at $\lambda_{ex} = 514$ nm. Lifetimes marked with a star symbol are larger than the maximum measured time delay and should therefore be regarded as infinite in the context of the performed measurements.

9 Temperature Dependence of Quantum Yields

In Fig. S15 the predicted and experimental fluorescence quantum yields as functions of the temperature are plotted as lines and crosses, respectively. In all solvents for which experimental data are available at more than one point, the quantum yield increases at lower temperatures. The largest temperature range could be investigated for CH_2Cl_2. For this solvent of intermediate polarity a quite good agreement between experiment and theory is found with an increase of the fluorescence quantum yield from 17 % at 293 K to 54% at 195 K. For the least polar solvent MCH a small increase from QY=1 % at T=293 K to 4 % at 248 K can be discerned in the experimental data. For the unpolar solvent MCH (where the dye aggregation precludes measuremnts below 248 K) and the polar solvent DMSO (where no measurements below 291 K are possible due to the solidification of the solvent) the inflection points lie below or above the temperature range that can be measured. Based on the position of the inflection point relative to room temperature, the solvents can be divided into three groups: the unpolar solvents MCH (and Tol) which suppress the fluorescence, the polar solvents DMSO (and MeCN) which enable fluorescence and the solvents CH_2Cl_2 (and $CHCl_3$), which allow to tune the fluorescence quantum yield of **DCBT** over a relatively narrow temperature interval close to room temperature.

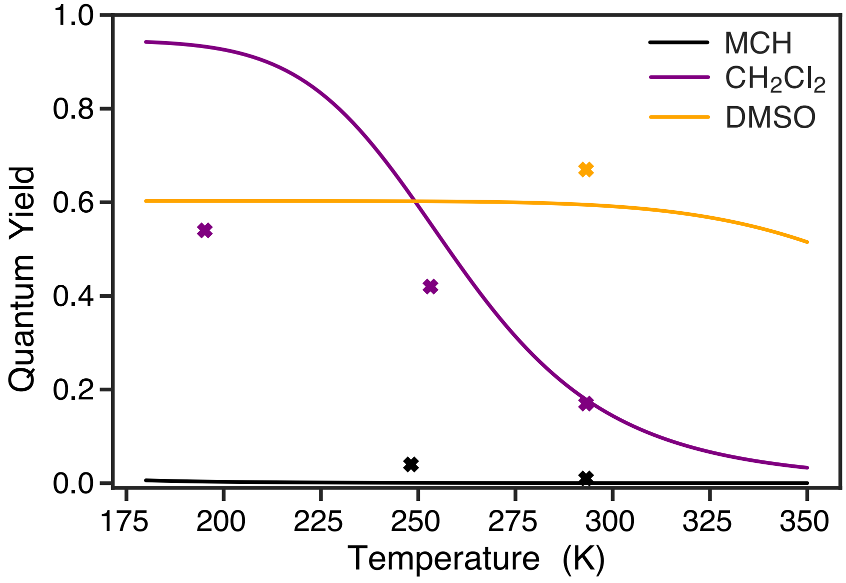

Fig. S15: Temperature dependence of the quantum yield in different solvents. Solid curves correspond to the predictions, crosses are measurements.

References

1 W. Liptay, *Dipole Moments and Polarizabilities of Molecules in Excited States*, Academic Press, 1974.

2 W. Baumann, *Physical Methods of Chemistry, Vol. 3B*, Wiley, 1989.

3 J. Buback, M. Kullmann, F. Langhojer, P. Nuernberger, R. Schmidt, W. F. and T. Brixner, *J. Am. Chem. Soc.*, 2010, **132**, 16510–16519.

4 F. Koch, A. Steinbacher, C. Consani, A. Zitzler-Kunkel, M. Stolte, F. Wrthner and T. Brixner, *Phys. Chem. Chem. Phys.*, 2016, **18**, 19820–19831.

5 X. Ma, M. Wenzel, H. Schmitt, M. Flock, E. Reusch, R. Mitric, I. Fischer and T. Brixner, *Phys. Chem. Chem. Phys.*, 2018, **20**, 15434–.

6 I. H. M. van Stokkum, D. S. Larsen and R. van Grondelle, *Biochim. Biophys. Acta - Bioenerg.*, 2004, **1657**, 82–104.

7 J. J. Snellenburg, S. P. Laptenok, R. Seger, K. M. Mullen and I. H. M. M van Stokkum, *J. Stat. Softw.*, 2012, **49**, 1–22.

8 K. M. Mullen and I. H. M. M van Stokkum, *J. Stat. Softw.*, 2007, **18**, 1–46.

9 A. Schfer, C. Huber and R. Ahlrichs, *J. Chem. Phys.*, 1994, **100**, 5829–5835.

10 M. J. Frisch, G. W. Trucks, H. B. Schlegel, G. E. Scuseria, M. A. Robb, J. R. Cheeseman, G. Scalmani, V. Barone, G. A. Petersson, H. Nakatsuji, X. Li, M. Caricato, A. V. Marenich, J. Bloino, B. G. Janesko, R. Gomperts, B. Mennucci, H. P. Hratchian, J. V. Ortiz, A. F. Izmaylov, J. L. Sonnenberg, D. Williams-Young, F. Ding, F. Lipparini, F. Egidi, J. Goings, B. Peng, A. Petrone, T. Henderson, D. Ranasinghe, V. G. Zakrzewski, J. Gao, N. Rega, G. Zheng, W. Liang, M. Hada, M. Ehara, K. Toyota, R. Fukuda, J. Hasegawa, M. Ishida, T. Nakajima, Y. Honda, O. Kitao, H. Nakai, T. Vreven, K. Throssell, J. A. Montgomery, Jr., J. E. Peralta, F. Ogliaro, M. J. Bearpark, J. J. Heyd, E. N. Brothers, K. N. Kudin, V. N. Staroverov, T. A. Keith, R. Kobayashi, J. Normand, K. Raghavachari, A. P. Rendell, J. C. Burant, S. S. Iyengar, J. Tomasi, M. Cossi, J. M. Millam, M. Klene, C. Adamo, R. Cammi, J. W. Ochterski, R. L. Martin, K. Morokuma, O. Farkas, J. B. Foresman and D. J. Fox, *Gaussian16 Revision B.01*, 2016.

11 V. Barone and M. Cossi, *J. Phys. Chem. A*, 1998, **102**, 1995–2001.

12 B. Mennucci, E. Cances and J. Tomasi, *J. Phys. Chem. B*, 1997, **101**, 10506–17.

13 J. Tomasi, B. Mennucci and E. Cances, *J. Mol. Struct.*, 1999, **464**, 211–26.

14 E. Cances, B. Mennucci and J. Tomasi, *J. Chem. Phys.*, 1997, **107**, 3032–41.

15 M. Cossi and V. Barone, *J. Chem. Phys.*, 2001, **115**, 4708–17.

16 F. Santoro, R. Improta, A. Lami, J. Bloino and V. Barone, *The Journal of Chemical Physics*, 2007, **126**, 084509.

17 F. Duschinsky, *Acta Physicochim. URSS*, 1937, **7**, 551–577.

18 M. J. Bearpark, M. A. Robb and H. Bernhard Schlegel, *Chemical Physics Letters*, 1994, **223**, 269–274.

19 B. O. Roos, *Adv. Chem. Phys.*, 1987, **69**, 399.

20 H.-J. Werner and P. J. Knowles, *J. Chem. Phys*, 1985, **82**, 5053.

21 T. Busch, A. D. Esposti and H. Werner, *J. Chem. Phys.*, 1991, **94**, 6708–6715.

22 P. J. Knowles and H.-J. Werner, *Chem. Phys. Lett.*, 1985, **115**, 259 – 267.

23 T. Shiozaki, *Wiley Interdisciplinary Reviews: Computational Molecular Science*, 2018, **8**, e1331.

24 Q. Wu and T. Van Voorhis, *The Journal of Physical Chemistry A*, 2006, **110**, 9212–9218.

25 Q. Wu and T. Van Voorhis, *Journal of Chemical Theory and Computation*, 2006, **2**, 765–774.

26 Y. Shao, Z. Gan, E. Epifanovsky, A. T. Gilbert, M. Wormit, J. Kussmann, A. W. Lange, A. Behn, J. Deng, X. Feng, D. Ghosh, M. Goldey, P. R. Horn, L. D. Jacobson, I. Kaliman, R. Z. Khaliullin, T. Ku, A. Landau, J. Liu, E. I. Proynov, Y. M. Rhee, R. M. Richard, M. A. Rohrdanz, R. P. Steele, E. J. Sundstrom, H. L. Woodcock, P. M. Zimmerman, D. Zuev, B. Albrecht, E. Alguire, B. Austin, G. J. O. Beran, Y. A. Bernard, E. Berquist, K. Brandhorst, K. B. Bravaya, S. T. Brown, D. Casanova, C.-M. Chang, Y. Chen, S. H. Chien, K. D. Closser, D. L. Crittenden, M. Diedenhofen, R. A. DiStasio, H. Do, A. D. Dutoi, R. G. Edgar, S. Fatehi, L. Fusti-Molnar, A. Ghysels, A. Golubeva-Zadorozhnaya, J. Gomes, M. W. Hanson-Heine, P. H. Harbach, A. W. Hauser, E. G. Hohenstein, Z. C. Holden, T.-C. Jagau, H. Ji, B. Kaduk, K. Khistyaev, J. Kim, J. Kim, R. A. King, P. Klunzinger, D. Kosenkov, T. Kowalczyk, C. M. Krauter, K. U. Lao, A. D. Laurent, K. V. Lawler, S. V. Levchenko, C. Y. Lin, F. Liu, E. Livshits, R. C. Lochan, A. Luenser, P. Manohar, S. F. Manzer, S.-P. Mao, N. Mardirossian, A. V. Marenich, S. A. Maurer, N. J. Mayhall, E. Neuscamman, C. M. Oana, R. Olivares-Amaya, D. P. ONeill, J. A. Parkhill, T. M. Perrine, R. Peverati, A. Prociuk, D. R. Rehn, E. Rosta, N. J. Russ, S. M. Sharada, S. Sharma, D. W. Small, A. Sodt, T. Stein, D. Stck, Y.-C. Su, A. J. Thom, T. Tsuchimochi, V. Vanovschi, L. Vogt, O. Vydrov, T. Wang, M. A. Watson, J. Wenzel, A. White, C. F. Williams, J. Yang, S. Yeganeh, S. R. Yost, Z.-Q. You, I. Y. Zhang, X. Zhang, Y. Zhao, B. R. Brooks, G. K. Chan, D. M. Chipman, C. J. Cramer,

W. A. Goddard, M. S. Gordon, W. J. Hehre, A. Klamt, H. F. Schaefer, M. W. Schmidt, C. D. Sherrill, D. G. Truhlar, A. Warshel, X. Xu, A. Aspuru-Guzik, R. Baer, A. T. Bell, N. A. Besley, J.-D. Chai, A. Dreuw, B. D. Dunietz, T. R. Furlani, S. R. Gwaltney, C.-P. Hsu, Y. Jung, J. Kong, D. S. Lambrecht, W. Liang, C. Ochsenfeld, V. A. Rassolov, L. V. Slipchenko, J. E. Subotnik, T. Van Voorhis, J. M. Herbert, A. I. Krylov, P. M. Gill and M. Head-Gordon, *Molecular Physics*, 2015, **113**, 184–215.

27 J. A. Van Allan and G. A. Reynolds, *J. Heterocycl. Chem.*, 1980, **17**, 1585–1586.

28 A. Levitz and S. T. Ladani, *Dyes Pigments*, 2014, **105**, 238–249.

29 Y.-S. Yao, Y.-T. Chuai, X.-S. Wang and B.-W. Zhang, *Chinese J. Chem.*, 2006, **24**, 1137–1143.

30 D. Venkatakrishnarao, Y. S. L. V. Narayana, M. A. Mohaiddon, E. A. Mamonov, N. Mitetelo, I. A. Kolmychek, A. I. Maydykovskiy, V. B. Novikov, T. V. Murzina and R. Chandrasekar, *Adv. Mater.*, 2017, **29**, 1605260.

31 A. Corney, *Atomic and Laser Spectroscopy*, Clarendon Press, Oxford, 2006.

32 S. H. Lin, *Theor. Chem. Act.*, 1968, **10**, 301–310.

33 Q. Peng, Y. Yi, Z. Shuai and J. Shao, *J. Am. Chem. Soc.*, 2007, **129**, 9333–9339.

34 S. Banerjee, A. Baiardi, J. Bloino and V. Barone, *J. Chem. Theor. Comput.*, 2016, **12**, 774–786.

35 G. Schatz and M. Ratner, *Quantum Mechanics in Chemistry*, Dover, 1993.

36 R. Englman and J. Jortner, *Mol. Phys.*, 1970, **18**, 145–164.

37 K. Huang and A. Rhys, *Proc.Roy. Soc. Lond*, 1950, **A204**, 406–423.

38 R. O'Rourke, *Physical Review*, 1953, **91**, 265.

39 C. Cohen-Tannoudji, B. Diu and F. Lalo, *Quantenmechanik*, Wiley-VCH, 1991, vol. 2.

40 K. Pearson, *Proc. R. Soc. Lond. A*, 1895, **58**, 240–242.

41 *Microkat Solvent Datasheet: Methylcyclohexane*, `http://www.microkat.gr/msdspd90-99/Methylcyclohexane.html`, accessed on Sept. 12, 2019.

42 *National Center for Biotechnology Information. PubChem Database. Methylcyclohexane, CID=7962*, `https://pubchem.ncbi.nlm.nih.gov/compound/Methylcyclohexane`, accessed on Sept. 12, 2019.

43 *Anton Paar Wiki*, `https://wiki.anton-paar.com/en/toluene/`, accessed on Sept. 12, 2019.

44 *National Center for Biotechnology Information. PubChem Database. Toluene, CID=1140*, https://pubchem.ncbi.nlm.nih.gov/compound/Toluene, accessed on Sept. 12, 2019.

45 *National Center for Biotechnology Information. PubChem Database. Chloroform, CID=6212*, https://pubchem.ncbi.nlm.nih.gov/compound/Chloroform, accessed on Sept. 12, 2019.

46 *National Center for Biotechnology Information. PubChem Database. Dichloromethane, CID=6344*, https://pubchem.ncbi.nlm.nih.gov/compound/Dichloromethane, accessed on Sept. 12, 2019.

47 *National Center for Biotechnology Information. PubChem Database. Acetonitrile, CID=6342*, https://pubchem.ncbi.nlm.nih.gov/compound/Acetonitrile, accessed on Sept. 12, 2019.

48 *National Center for Biotechnology Information. PubChem Database. Chloroform, CID=6212*, https://pubchem.ncbi.nlm.nih.gov/compound/Dimethyl-sulfoxide, accessed on Sept. 12, 2019.

49 L. Onsager, *J. Am. Chem. Soc.*, 1936, **58**, 1486–1493.

50 A. Milischuk and D. V. Matyushov, *J. Phys. Chem. A*, 2002, **106**, 2146–2157.
